# Molecular Heptazine–Platinum Complexes to Model Metal–Support Coordination in Platinized Graphitic Carbon Nitride: Insights into Catalyst Stability and Support Suitability

**DOI:** 10.1021/acs.inorgchem.6c04290

**Published:** 2026-09-17

**Authors:** Chi-Feng Lu, Tsu-Lien Hung, Jiliang Zhang, Vincent Wing-hei Lau

**Affiliations:** † Department of Chemistry, 34912 National Cheng Kung University, No. 1 University Road, Tainan City 701, Taiwan (ROC); ‡ Core Facility Center, National Cheng Kung University, No. 1 University Road, Tainan City 701, Taiwan (ROC); § School of Materials Science and Engineering, 74546 Dalian Jiaotong University, Dalian 116028, P. R. China

## Abstract

Solid support materials are crucial in heterogeneous catalysis for stabilizing active metallic species. Among potential support materials, heptazine-based “graphitic carbon nitride” or “gC_3_N_4_” has received significant research attention due to its abundant nitrogen sites, which are widely hypothesized to anchor catalytic metals through direct coordination. To better understand such a complexation mode while circumventing the structural complexity of gC_3_N_4_ materials, we employed as molecular models the heptazine compounds melem, cyamelurate, and melonate, with which we attempted Pt–heptazine complexation to emulate the interactions between the terminations and defects of gC_3_N_4_ with platinum, an important catalytic species. Despite extensive characterizations, only second-sphere coordination could be verified, while primary-sphere platinum-heptazine complexation could not be unambiguously observed, suggesting that such interactions may be intrinsically weak under the investigated conditions. These findings from model systems imply that gC_3_N_4_ materials may exhibit weaker-than-expected coordinative stabilization. Consistent with this implication, a series of Pt–gC_3_N_4_ composites evaluated as catalysts in nitrobenzene transfer hydrogenation exhibited platinum leaching more severely compared to a benchmark Pt–TiO_2_. Beyond providing molecular-level insights into gC_3_N_4_ supports, the methodology of the model compound presented here offers a useful framework for investigating interfacial coordinative interactions in support materials.

## Graphical abstract

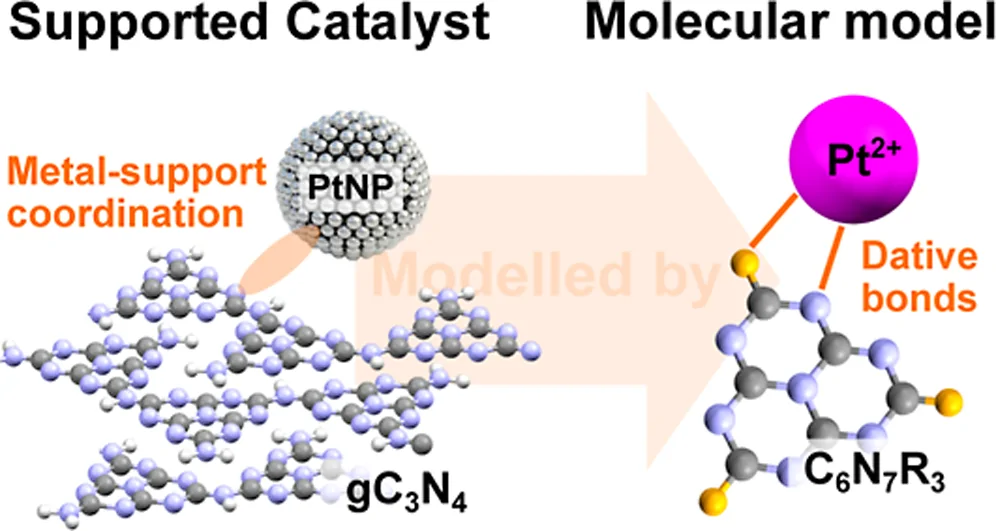

## Introduction

Heterogeneous noble-metal catalysts are employed for a variety of important chemical reactions, especially hydrogenation, exhaust catalysis (i.e., CO and hydrocarbon oxidation and reductive deNOx), various transformation reactions (e.g., isomerization, cross-coupling, and reforming), as well as cocatalysts in electro/photocatalytic systems.^1–4^ Due to their cost and rarity, noble metals are often miniaturized into nanoparticles or even single-atom catalysts and deposited over another solid, the support. Interfacial phenomena between the metal and the support, collectively referred to as metal–support interactions (MSI), play a fundamental role in immobilizing the catalytic species, maintaining high dispersion, and preventing the loss of active surfaces from aggregation.^2,5–7^ Moreover, the support may be noninnocent and could modify the catalytic activity and/or selectivity through MSI-related effects.^5,8,9^ For these reasons, the development of suitable support materials is crucial to economize the use of noble-metal catalysts. One class of materials proposed as catalyst support is “graphitic carbon nitride” or gC_3_N_4_, a misnomer that describes heptazine-based condensed phases like those depicted in Figure 1a and b, which have been extensively investigated due to their photocatalytic reactivity for solar fuel production and organic transformations,^10^ as well as potential applicability as catalyst support, notably for single-/few-atom catalysts (SAC).^11,12^

**Figure 1:**
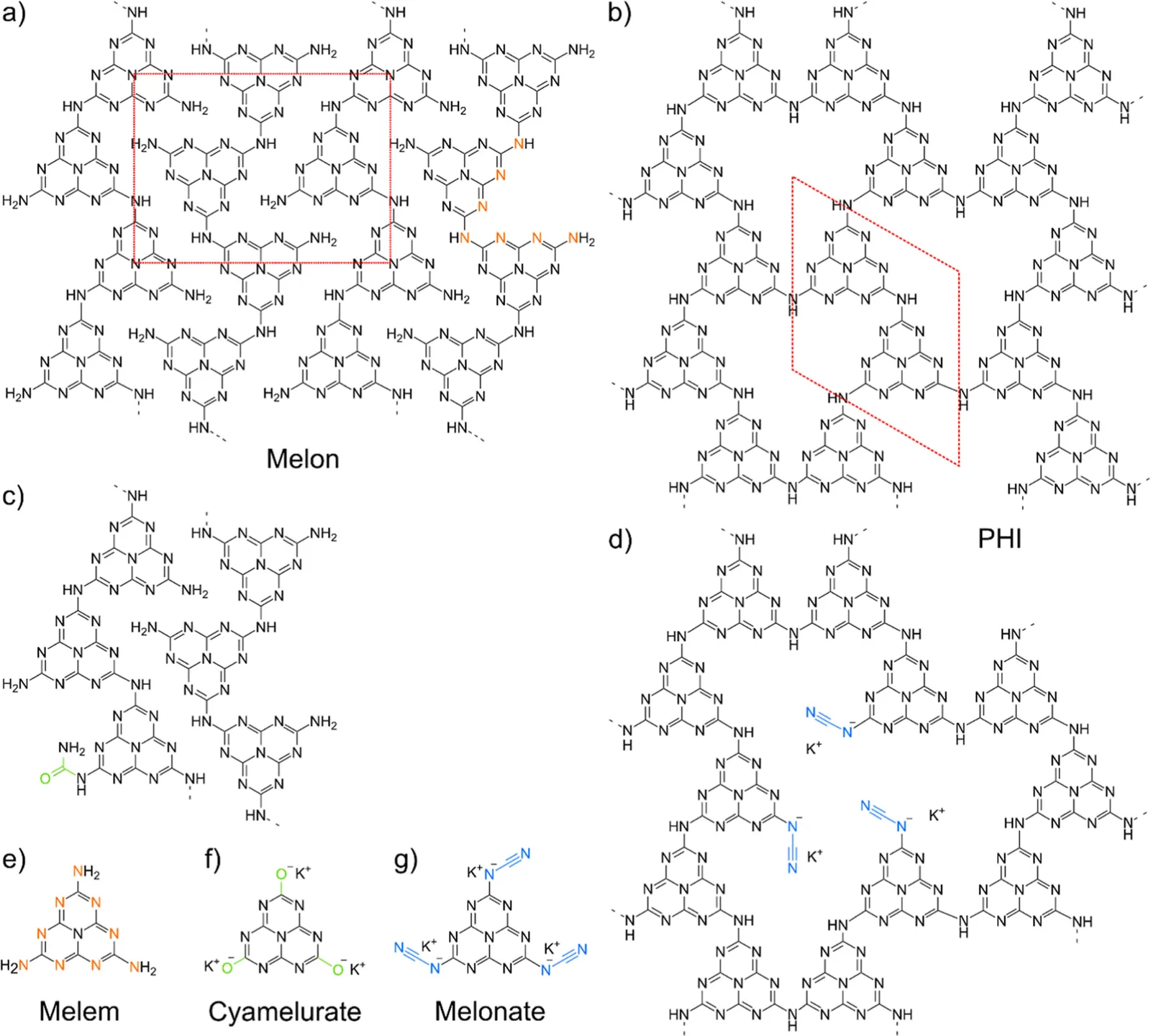
Two-dimensional structure determined from spectroscopy and diffractometry of (a) melon,^13^ where the nitrogens (some drawn in orange) are presumed to be coordination sites, and (b) poly­(heptazine imide), PHI.^14^ The red dashed enclosures in (a,b) indicate the plane cell. Hypothesized structures of (c) melon with a urea defect in green and (d) PHI with three cyanamide moieties as a point defect in blue; (e–g) structures of the three heptazine-based molecules modeling melon and PHI, with the functional groups intended to emulate defects and terminations color-coded identically to panels a, c, and d.

Focusing on their application as support, the aromatic sp2 nitrogen and primary/secondary amines (colored orange in Figure 1a) in gC_3_N_4_ are widely thought to be capable of coordinative stabilization of metal centers.^12,15–17^ However, these nitrogen sites have poor Lewis basicity given that heptazine is electron withdrawing,^18^ and such coordination mode has not been unambiguously demonstrated. Alternatively, coordination with noble metals could occur through the crystallographic defects inherent to gC_3_N_4_ materials, defects which are proposed to occur through incomplete transformation of the gC_3_N_4_ precursors. As one case in point, the archetype gC_3_N_4_ material melon,^13^ a 1D heptazine-based polymer linked by 2° amines drawn in Figure 1a, has been shown to have oxygen-bearing functional groups (colored green in Figure 1c) if synthesized from urea.^19^ Residual functional groups in melon arising from incomplete condensation and cyclization of other precursors, even those without oxygen, have been proposed based on results from mass spectroscopy.^20,21^ Another case is point is poly­(heptazine imide), or PHI, a 2D heptazine-based array comprising heptazines conjoined also by 2° amines (Figure 1b).^14^ PHI often contains the functional group cyanamide (−NCN^–^) as point defects or surface termination, as colored in blue in Figure 1d.^14,20^ While these defect-associated groups may be the likelier coordination sites, this remains to be unambiguously demonstrated, and acquiring a better understanding of their coordination mode and complexation strength with noble metals could direct future development of this class of materials for catalyst support through defect engineering.

One challenge in investigating the interface between noble metals and gC_3_N_4_ materials is their structural heterogeneity, especially melon and PHI, which are typically synthesized under kinetic control, thereby resulting in the aforementioned structural defects from incomplete precursor cyclization/condensation. To facilitate their analyses, we take inspiration from the methodology of employing model compounds for studying real (commercial) solid catalysts.^22–26^ In this methodology, a model system that is structurally simpler but unambiguous is employed to emulate key features of more complex chemical systems. Although the model cannot replicate all features of the real system due to this structural simplification, this approach has been successful in eliciting fundamental insights, especially in elucidating the relationship between specific structural features and (catalytic) reactivity. Drawing parallels to the gC_3_N_4_ as molecular materials, we propose to model their surficial interaction with noble metals using heptazine-based small molecules, specifically those of Figure 1e–g that have systematic variations of the functional group on the heptazine’s 2,5,8-positions. Specifically, these functional groups structurally emulate the defects and terminations thought to be present in the parent material: melem^27^ with −NH_2_ to model the defect-free one-dimensional heptazine-based polymer melon (Figure 1a), melonate^28^ with −NCN^–^ to model the cyanamide defect often found in PHI (Figure 1d), and cyamelurate^29^ with −O^–^ to model oxygen-containing defects (Figure 1c). This methodology has previously been successful in identifying −NCN^–^ as a catalytically relevant structural feature, an insight that enabled the rational design of a heptazine-based catalyst having an order-of-magnitude increase in reaction kinetics.^20^ In this work, we will investigate the complexation between these heptazine molecules and platinum, considering not only this metal’s importance in catalysis^30,31^ but also the widespread use of Pt–gC_3_N_4_ in photocatalytic systems for H_2_ evolution.^32^ Given that metal–nitrogen coordination is considered to be central to gC_3_N_4_’s ability to stabilize metal catalysts as mentioned above, our focus will be on employing the heptazine molecular models to elicit insights into the Pt–heptazine complexation mode and coordination bond strength. As will be shown here, direct platinum–heptazine interactions could not be detected between the metal and these model compounds under the conditions tested, which could imply that metal–gC_3_N_4_ interactions may be weaker-than-expected for stabilizing metal catalysts. This implication was further tested by employing a series of Pt–gC_3_N_4_ as catalysts for transfer hydrogenation, where they suffered from metal leaching much more severely compared to a benchmark Pt–TiO_2_.

## Results and Discussion

In typical procedures for preparing supported platinum catalysts, precursors such as [(NH_3_)_4_Pt]^2+^, [PtCl_4_]^2–^ or Pt­(NO_3_)_*x*_ are solubilized and mixed or impregnated into the support material, followed by reduction to Pt(0) by the addition of a reductant or by heating under a reducing atmosphere, as illustrated schematically in Figure 2a. From the nucleation–growth theory, the platinum precursor undergoes heterogeneous nucleation to form metallic platinum as the catalytic species. If there is strong interaction between the metal precursor and the support, fewer mobile monomers are available for particle growth, resulting in nanoparticles. Moreover, when strong coordinative binding is present between the support and the metal (represented as red dashed lines in Figure 2b), platinum species with nonzero valency (gray in Figure 2b) have been observed even after harsh reductive treatment despite platinum’s propensity to reduce to the metallic state.^33–35^ To account for these observations, we model the Pt–gC_3_N_4_ interactions by examining the complexation between Pt^2+^ and the three heptazine-based molecular models, as graphically summarized in Figure 2c. The sections below are separated by the heptazine molecules employed in complexation with three Pt^2+^ precursors, [(NH_3_)_4_Pt]^2+^, [PtCl_4_]^2–^, or Pt­(NO_3_)_2_. For referencing, these three Pt precursors were first characterized by X-ray diffraction (XRD), as well as Fourier-transform infrared (FTIR) and X-ray photoelectron spectroscopy (XPS), and the results are shown in Figures S1 and S2 and accompanied by supporting discussions.

**Figure 2:**
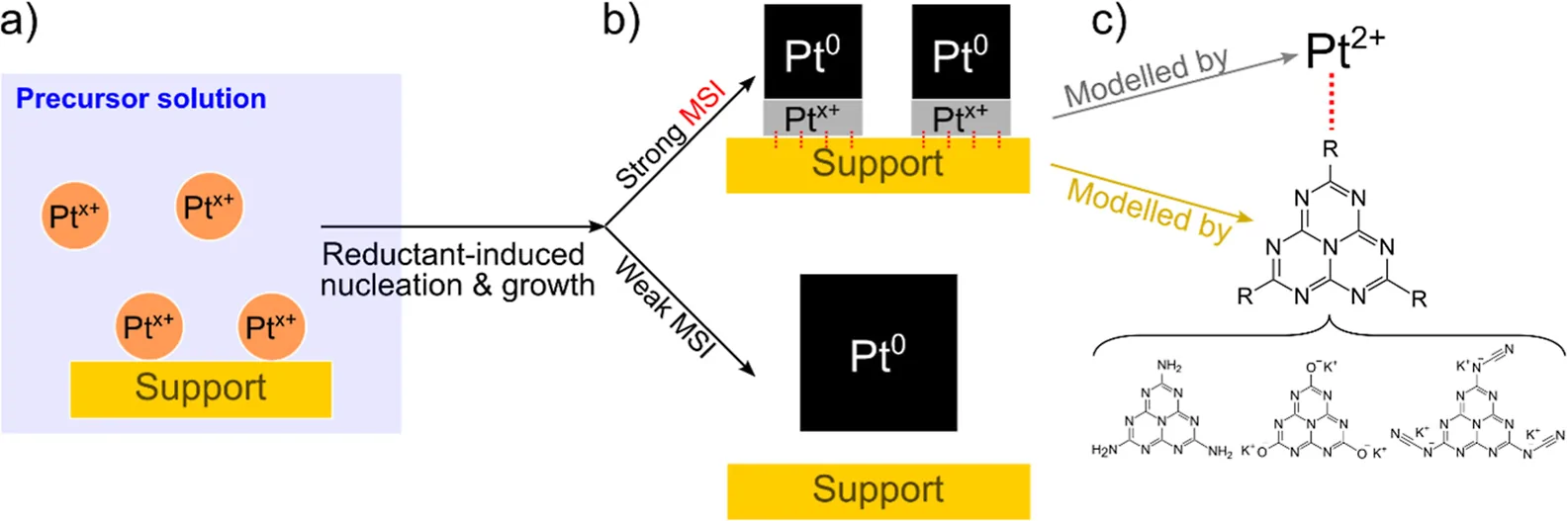
Experimental considerations in modeling metal–support interactions: (a) Depiction of heterogeneous nucleation and growth of Pt particles over support; (b) depiction of Pt particles with weak (bottom) and strong (top) coordinative interactions with the support, the latter represented by the red dashed lines, while Pt species with nonzero valency drawn in gray, which is (c) modeled in this work by the complexation between Pt^2+^ and the three heptazine molecular models.

### Cyamelurate—Modeling Oxygen-Bearing Defects

Based on previously published structure solutions of cyamelurate complexes,^36,37^ the metal is coordinated by the oxo anion solely or together with the outer heptazine nitrogens in a bidentate mode. Since the oxo anions are located on opposite sides of the heptazine core, we expect metal–cyamelurate complexation to lead to 1D/2D coordination chains/arrays that have decreased solubility. In other words, upon mixing a solution of the metal precursor with a solution of this heptazine molecule, we anticipate a precipitate should complexation occur. However, in our preliminary tests using aqueous solutions of the three platinum precursors, only tetraammineplatinum­(II) led to a product poorly soluble in water when mixed with a solution of potassium cyamelurate. Following optimization of crystal growth, we obtained crystals suitable for single crystal X-ray diffraction (SC-XRD) by acetonitrile vapor diffusion into a dilute aqueous mixture of potassium cyamelurate and [(NH_3_)_4_Pt]­(NO_3_)_2_. The structure (referred to as compound **1**) was solved and refined based on the SC-XRD data, and the crystallographic details are tabulated in Tables S2 and S3.

Compound **1**, the unit cell of which is drawn in Figure 3, has the space group *P*2_1_/*m* (no. 11) with the formula C_6_H_12_N_11_O_3_Pt. The platinum atom resides on a mirror site and adopts a square-planar coordination with four NH_3_ ligands. The Pt(1) site is well-defined and occupied at 92(2)%, while a minor degree of disorder is located at the Pt­(1A) and Pt­(1B) sites, which have been refined with occupancies of 4.1(4)% and 3.9(3)%, respectively, and are drawn with transparency in Figure 3a,b. Evidently, cyamelurate does not coordinate to platinum in the first coordination sphere but only in the second through hydrogen bonding with the NH_3_ ligands, as illustrated in Figure 3b. As annotated by the red and blue dashed lines, the cyamelurate’s oxo anion is separated by 2.93 and 3.23 Å with two of the nitrogens of the NH_3_ ligands, while the brown dashed line indicates the N···N separation of 3.25 Å between the cyamelurate nitrogen and the nitrogen of one of the NH_3_ ligands. Reminiscent of other second coordination shell complexes of cyamelurates,^29,38^ this structure thus comprises a hydrogen-bonded network of cyamelurates and diaquatetraammineplatinum complexes in the b/c-directions. Along the a-direction, a separation of 3.67 Å, as annotated in Figure 3c,d, refers to both π-stacking of the cyamelurate molecules and the stacking of square-planar platinum complexes owing to interactions between the 5d_*z*_^2^ and 6p_*z*_ orbitals of adjacent Pt centers.^39^ Both stackings have a slight offset that is more clearly shown when viewed along the *c*-direction, as depicted in Figure 3d. Here, the stacking distance and the offset are consistent with similar compounds. For the cyamelurate as an example, rubidium cyamelurate has a π–π stacking separation of 3.67 Å,^29^ while stacking distances in the range of 3.24–3.71 Å have been reported for Pt–Pt separation in offset stacks of platinum square-planar complexes.^39^

**Figure 3:**
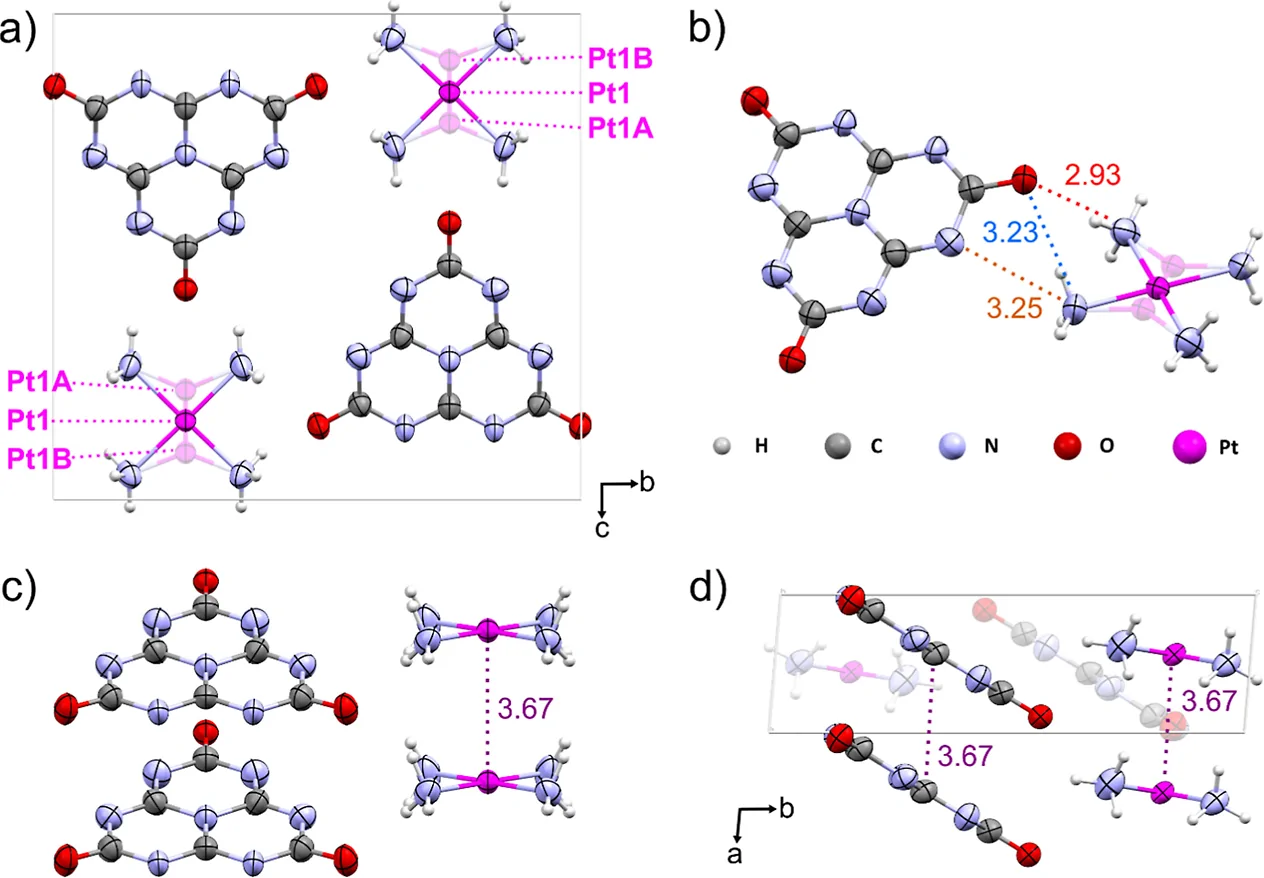
ORTEP diagram of compound **1** drawn at 50% probability: (a) unit cell viewed along the a-direction; (b) asymmetric unit showing the interatomic distances between the cyamelurate anion and the square-planar tetraammineplatinum complex. For (a,b), positional disorder of the platinum is represented by depicting platinum positions with low occupancies in increased transparency. (c,d) Interatomic distances as related to the π–π stacking of the cyamelurate molecules and the stacking of the square planar Pt complexes. All annotations of interatomic distances are in Ångstroms.

Compound **1** was also characterized by CNH elemental analysis (Table S4), which yielded a formula of C_6_N_11_H_15_ when normalized to 6 carbons. While the C/N ratio is consistent with the crystallographic formula, a larger-than-expected quantity of hydrogen was detected and could originate from measurement uncertainty arising from residual moisture or protons unaccounted for in the crystal structure. One explanation is suggested by XPS, which was performed to determine the oxidation state of the platinum. The spectra are shown in Figure S3, and the fitting parameters are summarized in Table S5. Focusing on the Pt 4f region, the 4f7/2 signal of **1** has a binding energy (BE) of 73.3 eV, which is within 0.1 eV to that of Pt­(NH_3_)_4_(NO_3_)_2_ at 73.2 eV (Table S1), signifying that both have the same platinum valency of 2+. Note that the XPS peak shift of ≤0.2 eV has sometimes been considered in the literature as an evidence of MSI,^40–42^ although we argue that a shift of such magnitude is unlikely to be significant given that the typical spectral resolution of commercial XPS with Al Kα X-ray is around 0.5 eV. For charge balance of the Pt^2+^, one of three oxo-anions of each cyamelurate must be protonated, which could partially account for the larger-than-expected amount of hydrogen in CHN elemental analyses. Fourier-transform infrared (FTIR) spectroscopy was thus performed to identify such protonation, and compound **1**’s spectrum is shown in Figure S4, together with those of the precursors as well as cyameluric acid. For reference, the dominant tautomer of cyameluric acid is depicted in the far right of Figure S4, where the protons reside at the heptazine nitrogen rather than on the oxo-anion (i.e.,–NH– rather than −OH),^43–45^ resulting in distinct IR bands. Specifically, the IR band of cyameluric acid at 1284 cm^–1^ can be assigned to C–NH–C,^46^ although this overlaps with the NH_3_/NO_3_^–^ bands of the platinum precursor. Nevertheless, two bands unique to cyameluric acid are observed in compound **1**: the small band at around 3500 cm^–1^ assignable to NH and the yet-to-be-assigned band at 900 cm^–1^.^47^ Determining the crystallographic position of this proton would be beyond the scope here, given that detailed study by nuclear magnetic resonance (NMR) spectroscopy is required.^43^ Nevertheless, in spite of this unresolved structural aspect pertaining to hydrogen positions, the crystal structure of **1** reveals that there is no direct coordination bonding between cyamelurate and platinum, suggesting that cyamelurate has insufficient Pt–cyamelurate complexation strength to displace any of the NH_3_ ligands on the Pt. Considering the exceptional stability of [(NH_3_)_4_Pt]^2+^ against ligand substitution,^48^ we next explored platinum complexes with more labile ligands in our attempts to form Pt–cyamelurate complexes, namely [PtCl_4_]^2–^ and Pt­(NO_3_)_2_.

### Attempted Complexation between Cyamelurate and K_2_PtCl_4_ and Pt­(NO_3_)_2_

For the other two platinum precursors, K_2_PtCl_4_ and Pt­(NO_3_)_2_, mixing aqueous solutions of these with potassium cyamelurate did not yield any precipitate. These results suggest that a (poorly soluble) coordination chain did not form in spite of the more labile chloro and nitrato ligands in the platinum precursors. Nevertheless, to examine whether complexation still occurred without complexation, we first analyzed the solid residue obtained by evaporating to dryness the aqueous mixture of potassium cyamelurate with either of these two Pt precursors. The characterization results by XRD, FTIR, and XPS are shown in Figures S5–S7. However, as discussed in the accompanying supplementary discussions, these results could not unambiguously demonstrate direct Pt–cyamelurate coordination in the solid residues. To probe potential complexation in the solution phase, we performed UV–vis titration experiments by tracking spectral variations across mixtures with varying ratios of cyamelurate with either of the two platinum precursors. As a widely accessible technique, UV–vis titration has been extensively applied for investigating solution-phase complexation and host–guest binding equilibria. In literature examples specifically for platinum complexes, a blue-shift of the UV–vis band has been observed for [PtBr_4–*x*_Cl_*x*_]^2–^ as the bromo ligand is progressively substituted for the more electronegative/electron-withdrawing chloro ligand,^49^ while peak-shift and/or intensity change has been reported for aqua-hydroxyl exchange^50^ and for acetylacetonate-amine/carboxylate exchange^51^ and light-induced pyridine-acetonitrile exchange.^52^ This technique is also employed for investigating platinum complexes’ interactions with DNA interactions based on the attenuation of UV–vis bands.^53–56^ Based on these literature precedents, we anticipate chloro/nitrato exchange in [PtCl_4_]^2–^ and Pt­(NO_3_)_2_ by the electron-withdrawing cyamelurate would result in the ground state stabilization manifested as a UV–vis blue shift. The spectra are presented in Figure S8, and as explained in detail in the accompanying supplementary discussions, none of the absorption bands exhibited discernible shift or intensity change (other than from concentration change), from which we inferred that this method failed to reveal coordinative interactions between cyamelurate and either K_2_PtCl_4_ or Pt­(NO_3_)_2_ even in the solution state, despite the lability of the chloro and nitrato ligands.

Combining the results from solid- and solution-state analyses, we infer that direct Pt–cyamelurate interactions are intrinsically weak under the evaluated conditions, or at least too weak to be detected by the analytical methods employed here. As cyamelurate serves as the molecular model for the oxygen-bearing defects of gC_3_N_4_, the results above could imply that such defects may not be the principal structural feature contributing to coordinative stabilization of the metal centers in Pt–gC_3_N_4_ composites, though this will be discussed further in the sections below. Nevertheless, as exemplified in the second-sphere coordination in compound **1**, these oxygen-based functional groups can facilitate intermolecular bonding with polar small molecules and could thus rationalize the increased catalytic reactivity observed for oxygenated gC_3_N_4_ on account of its stronger interactions with the substrate.^19^

### Melonate-Modeling the Cyanamide Defects

As the cyanamide moiety is known to coordinate to electron-rich metals,^57,58^ we expect melonate to also coordinate through the nitrogens of this functional group, perhaps together with the heptazine outer nitrogen(s). Such coordination modes are based on published structure solutions of complexes with first-coordination-sphere metal–melonate bonding.^28,59–61^ In these literature examples, chains of metal melonate are formed on account of the −NCN^–^ group being on opposite ends of the heptazine unit. However, similar to the cases of cyamelurate in the previous section, our preliminary tests using aqueous solutions of the three platinum precursors showed that only tetraammineplatinum­(II) formed a poorly soluble yet crystalline product with potassium melonate. After optimizing for crystal growth of this product, henceforth designated as compound **2**, crystals with a needle-like morphology were analyzed by SC-XRD, and the structure solution is shown in Figure 4. Further crystallographic details are tabulated in Table S8 and Table S9.

**Figure 4:**
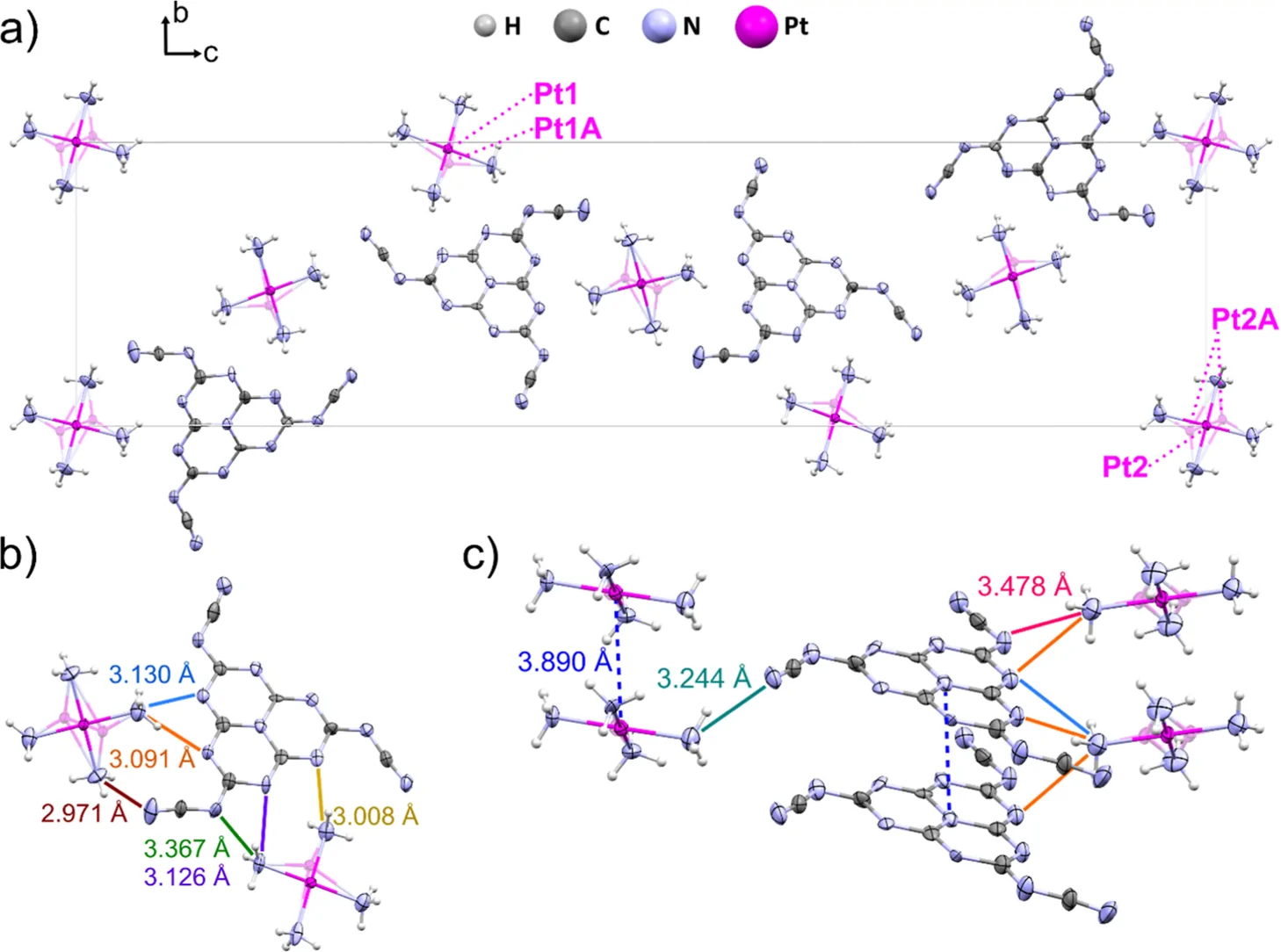
ORTEP diagram of compound **2** drawn at 50% probability: (a) one unit cell; (b) asymmetric unit showing the color-coded N–N interatomic distances; (c) stacking of square-planar [(NH_3_)_4_Pt­(II)] and of melonate. The blue dashed lines indicate the stacking distance, while other colored lines indicate the N–N interatomic distances arising from hydrogen bonding between [(NH_3_)_4_Pt­(II)] and melonate. All interatomic distances are in units of Ångstroms. In (b,c), the blue and orange lines refer to N–N interatomic distances as color-coded. Positional disorder of the platinum is represented by depicting platinum positions with low occupancies in increased transparency.

Compound **2** crystallizes in the space group *P*2_1_/*c* (number 14) with the formula C_18_H_36_N_38_Pt_3_, which is close to that determined by elemental analysis (Table S10) and would suggest the platinum to be Pt^2+^. This was confirmed by XPS (Figure S10), showing its Pt 4f_5/2_ signal to have a BE of 73.2 eV, identical to that of compounds **1** and [(NH_3_)_4_Pt­(II)]­(NO_3_)_2_. As is evident from the structure solution in Figure 4a, the metal remains complexed by four NH_3_ in square planar geometry in the first coordination sphere and melonate in the second. The structure is thus held by ionic and hydrogen bonding between tetraammineplatinum and melonate. Some hydrogen bonds in the second coordination sphere are shown in Figure 4b, where the color-coded annotations refer to the nitrogen–nitrogen distances, one nitrogen from the ligating NH_3_ and the other from melonate. Here, based on the short N–N separation of 2.97–3.37 Å, NH_3_ is inferred to hydrogen-bond to the amino and nitrile nitrogens of the cyanamide as well as the outer heptazine nitrogen. Stacking interactions between neighboring square-planar tetraammineplatinum and melonates are annotated with dashed blue lines in Figure 4c. Here, the stacking distance is relatively large at 3.890 Å, larger than those of compound **1** in Figure 3d, suggesting that close metal–metal and/or heptazine–heptazine stacking has been inhibited by the aforementioned hydrogen bonding between the NH_3_ and the melonate, some of which is also shown in this figure panel. As with the cyamelurate, both platinum atoms exhibit positional disorder at general and inversion sites. Such disorder was modeled in our structure by atomic positions having partial occupancies, which have been depicted with transparency in Figure 4. Similar to the disordering behavior in compound **1**, the occupancies of Pt(1) and Pt(2) sites reach 94.7(6)% and 92.9(8)%, respectively. However, disorder arising from thermal motion of these two platinum sites led to residual electron densities separated from the two metallic atoms by a distance below 2 Å that triggered Alert A/B in the CheckCIF. As detailed in the supporting discussions accompanying the SC-XRD results of compound **2**, we attribute such large thermal motion to the lack of strong intermolecular interactions stabilizing the platinum positions,^62^ especially in the stacking direction along the *a*-axis. It is thus evident of the structure of compound **2** that, as in the case for cyamelurate, melonate failed to displace the NH_3_ ligands despite the complexation ability of cyanamide that was mentioned above. The absence of Pt–melonate complexation in the first coordination sphere has also been corroborated in the FTIR spectrum of compound **2** (Figure S9), which shows negligible differences in the characteristic vibration bands corresponding to the cyanamide (2130–2170 cm^–1^) and heptazine core (797 cm^–1^) when compared to potassium melonate. Considering the stability of [(NH_3_)_4_Pt]^2+^ as mentioned above, we next focused on platinum–melonate complexation using the platinum precursors with more labile ligands, [PtCl_4_]^2–^ and Pt­(NO_3_)_2_.

### Attempted Complexation between Melonate and K_2_PtCl_4_ and Pt­(NO_3_)_2_

Similar to the cases of cyamelurate above, aqueous solutions of melonate mixed with either K_2_PtCl_4_ or Pt­(NO_3_)_2_ did not yield insoluble crystals, from which we inferred the failure to form coordination polymers that would otherwise render the complex insoluble. For further evidence that could support Pt-melonate complexation, we characterized the residues from evaporating aqueous mixtures of these two platinum precursors and potassium melonate to dryness by XRD, FTIR (Figure S11) and XPS (Figure S12). UV–vis titration was also performed to acquire evidence for complexation in the solution phase (Figure S13). However, as explained in the supplementary discussions accompanying these results, we did not observe conclusive evidence of Pt–melonate interactions based on the lack of distinct crystalline phases and the absence of spectral shifts or intensity change (beyond concentration-dependent effects). In other words, as in the case of the cyamelurates, we infer that direct Pt–melonate interactions are inherently weak under the conditions employed, or at least too weak to be detected by the analytical methods employed here. Since melonate acts as the molecular model for the –NCN^–^ defect often observed in PHI, these results imply that this functional group may have weaker-than-expected coordinative interactions for stabilizing the metal centers in Pt–gC_3_N_4_ composites, as detailed further in the sections below. Nevertheless, as suggested by second-sphere coordination in the crystal structure of **2** and analogous to the case of oxygen-based defects above, this ionic/polar defect could enable stronger intermolecular interactions that rationalize the correlation^14,20^ between the density of the cyanamide defect and the catalytic reactivity of PHI on account of their interactions with the reaction substrate.

### Melem-Modeling the Pristine Heptazine-based gC_3_N_4_ Termini

Of the three heptazine model molecules, only melem is poorly soluble in water. To our knowledge, only one publication^63^ has reported a crystal structure that conclusively evidenced the formation of a melem-metal complex. In this reference, the complex is that of silver, which is coordinated by melem through the heptazine nitrogen rather than the −NH_2_, a result that is consistent with the weak basicity of such amines (as explained in the introduction). By extension, we can infer the −NH_2_ and −NH– of melon and related gC_3_N_4_ to be likewise weak Lewis bases that are unlikely to coordinate to metals. Inspired by this work, we first attempted to form a Pt–melem complex by adapting the procedure in this publication, which involves suspending melem in an aqueous solution of the metal precursor for a certain duration. Afterward, the insoluble melem sample was recovered and characterized by XRD, FTIR, and XPS for the presence of Pt-melem complexation. The results, presented in Figures S14 and S15 and explained in detail in the accompanying supplementary discussions, are inconclusive as to the presence of Pt-melem coordination. Instead, the dominant phase observed was melem hydrate, and the platinum species present are likely [(NH_3_)_4_Pt]^2+^ or [PtCl_4_]^2–^ adsorbed onto the surface, or at least coordinated in the second sphere within melem hydrate’s network. The failure to form a detectable quantity of Pt–melem complex might be attributed to the poor solubility of melem in water. Thus, we have also attempted to prepare this complex in dimethyl sulfoxide (DMSO), one of the few solvents capable of dissolving melem. This solvent is strongly coordinating and is known to displace chloro ligand^64^ in K_2_PtCl_4_, although the NH_3_ ligand in [(NH_3_)_4_Pt^II^]­(NO_3_)_2_ is less likely to be substituted owing to its high complexation constant.^65^ Hence, we focused on potential complexation between melem and these two platinum salts upon mixing DMSO solutions of both and analyzed the solution phase for possible presence of Pt–melem coordination by UV–vis titration. Any precipitate formed from the DMSO solution was also characterized by XRD, FTIR, and XPS, the results of which are shown in Figures S16–S18. As detailed in the accompanying Supporting Information, we did not observe unambiguous evidence of Pt–melem complexation, suggesting that direct coordinative interactions between melem and platinum are intrinsically weak under the evaluated conditions. Considering that melem serves as a molecular model for the nitrogens of the heptazine core as well as the primary/secondary amine terminations in melon and in PHI, these results imply that such nitrogen sites may exhibit a weaker-than-expected coordinative affinity for platinum species. Whether such coordinative interactions from these nitrogen sites (or from the cyanamide or oxygen-based moieties) are sufficient for metal stabilization will be addressed in the next section.

### Evaluation of Model Compound Methodology and Implications for the Stability of the Pt–gC_3_N_4_ Catalytic System

In our evaluation of the three heptazine molecular models and three Pt^2+^ precursors above, we observed only second-sphere coordination or uncomplexed species, whereas direct primary-sphere Pt–heptazine coordination was not unambiguously ascertained under the tested conditions, suggesting that such direct coordinative interactions may be thermodynamically limited. Uncovering such interactions in the model systems might require exploring a broader experimental parameter space (e.g., alternative solvents, temperature, etc.) or deploying analytical techniques with exceptional sensitivity. As examples regarding the latter point, platinum-195 nuclear magnetic resonance spectroscopy is highly sensitive to changes in local coordination environment but suffers from poor receptivity (3.51 × 10^–3^ vs ^1^H), while synchrotron-based X-ray techniques and electron microscopies are likewise powerful but less accessible.^66,67^ Nevertheless, if Pt–heptazine interactions require highly specific conditions to arise or ultrasensitive techniques to be observed, one preliminary implication of our present results is that the gC_3_N_4_ family of materials (as modeled by the heptazine molecules herein) may not exhibit coordinative interactions that are sufficiently strong as to stabilize metallic species. Given that this work aims to model the coordinative interactions in gC_3_N_4_ using heptazine molecules, we next discuss the inherent limitations of the model methodology.

Considering that the methodology of model systems employs simpler but structurally unambiguous analogs to emulate specific features of complex systems, an inevitable outcome of this simplification is that the models cannot completely replicate all properties of the real systems, especially those arising from structural and morphological complexity (the so-called “material gap”). Specific to our heptazine models here, while they can emulate the monodentate coordinative ability of gC_3_N_4_ polymers and frameworks, they cannot capture the effects arising from polycondensation, such as shifts in optoelectronic properties and magnitude of dispersion forces. However, regarding the former, previous studies have demonstrated that heptazine polycondensates behave as “quasi-monomers”, whereby electron densities are largely localized within each heptazine monomer (at least in the in-plane direction) owing to the high energy barrier of the bridging 2° amines, such that optoelectronic properties differ only subtly when comparing monomers to linear oligomers and polymers.^68–70^ In contrast, electron delocalization in the stacking directions has been proposed in melon and PHI, an effect that these heptazine molecules cannot model.^71–73^ However, for the heptazine-based melon and PHI, this through-plane electron delocalization reduces local electron density, in turn attenuating the coordination bond strength of the donor nitrogen sites (analogous to how quinoline, with its greater electron delocalization, reportedly forms a weaker metal coordination bond compared to pyridine^74^). Hence, compared to their polycondensed counterparts (i.e., melon and PHI), heptazine model molecules would have a similar or greater tendency toward metal coordination considering their relatively localized electron density, though they have weaker dispersion interactions owing to their smaller molecular sizes.

Another limitation to the model methodology relates to the higher conformational degrees of freedom in the model molecules as compared to the heptazine units in polycondensates that are constrained by covalent bonding, π–π stacking, and morphological effects.^75^ For the model molecules, due to the increased degrees of freedom and/or flexibility, they can adopt the conformation most conducive to platinum coordination, in turn artificially increasing their ability for metal coordination. In contrast, considering that the model compounds were investigated in solutions, competing interactions such as solvation could conversely artificially lower the ability of these model molecules to form metal complexes. Nevertheless, considering that complexation requires the ligand to displace the species surrounding the metal, including the (coordinating) solvent, if Pt–heptazine complexation is readily outcompeted by other interactions such as solvation, these Pt–heptazine interactions may be relatively weak. Lastly, our monomeric heptazine models cannot undergo multidentate coordination from conjoined heptazines present in the polymer/framework, such as in poly­(tri/heptazine imide)^14,76^ and in Zn-melam, a dimer melamine coordinated to Zn^2+^,^46^ which are depicted in Scheme 1. Since the multidentate complexation constant (β_overall_) is the cumulative product of sequential stepwise coordination (β_overall_ = β_1_ × β_2_ ×···), the heptazine molecules employed herein can only model the process corresponding to β_1_. Considering the geometric freedom and lack of electron delocalization as mentioned above, we would expect these heptazine molecules to exhibit inflated values of β_1_ when compared to their polycondensed counterparts. However, the absence of detectable monodentate coordination here would imply that the value of β_1_ may be small, thereby imposing an upper bound to β_overall_, in turn implying that gC_3_N_4_ materials may have weaker-than-expected coordination ability.

**Scheme 1:**
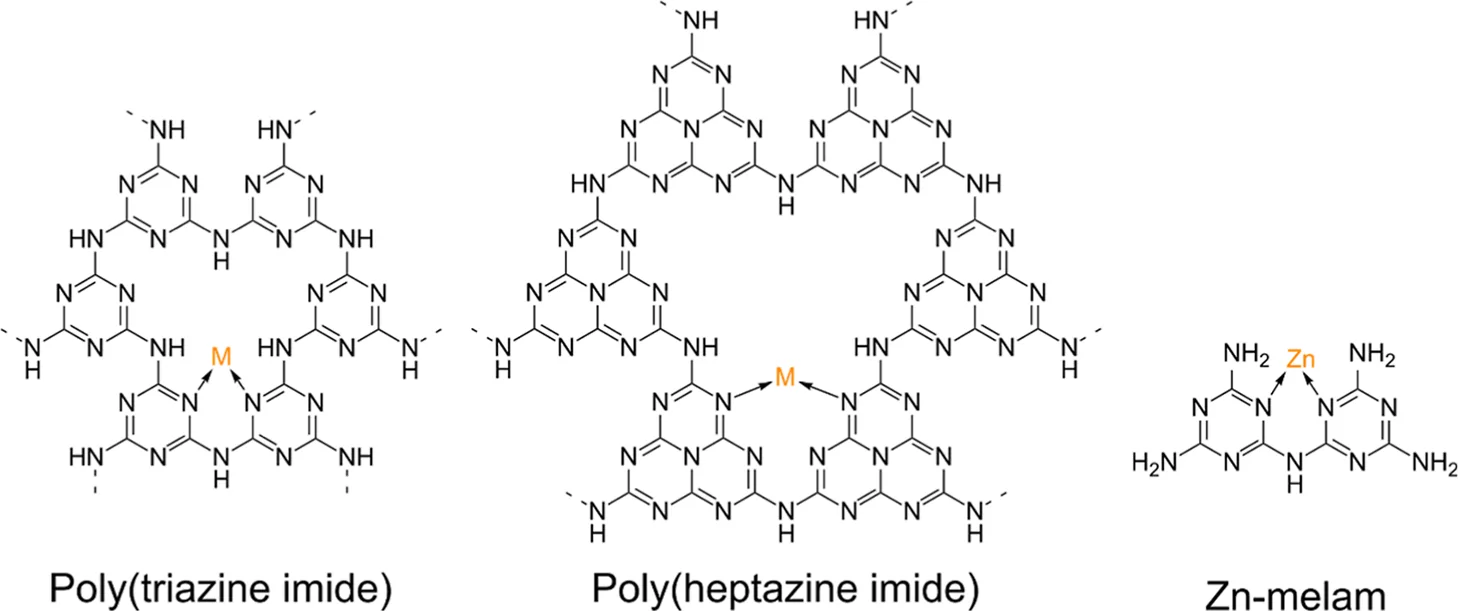
Depiction of the Multidentate Complexation of Metal (Orange “M”) by Poly­(tri-/heptazine Imide) and by Melam, a Dimer of Melamine

Although the heptazine molecules employed herein can emulate the monodentate coordinative interactions in gC_3_N_4_, these model systems cannot be expected to fully replicate all phenomena arising from polycondensations as raised in the preceding discussions. Consequently, while the inability to unambiguously confirm direct Pt–heptazine coordination in our model studies implies that the gC_3_N_4_ family of materials has a weaker-than-expected ability to coordinate to metal centers, macroscopic validation will be necessary to determine whether the findings from the molecular heptazine molecules can be translated to gC_3_N_4_ polycondensates. This validation is especially crucial considering that the implications derived from our model studies contrast the oft-claimed advantage of gC_3_N_4_ materials over carbon-based supports, that the abundant nitrogens enable metal stabilization through coordination interactions as mentioned in the introduction. Hence, to empirically test whether gC_3_N_4_ could prevent mobility/detachment of supported metallic catalyst through such coordinative stabilization, we subsequently tested a series of Pt–gC_3_N_4_ catalysts, focusing on quantifying metal leaching after a catalytic reaction. Here, the series of Pt–gC_3_N_4_ employed are Pt on melon prepared from melamine as the archetype combination (designated Pt–Melon-M),^32,77^ Pt on melon prepared from urea that has oxygen-bearing groups, as mentioned above (Pt–Melon-U),^19^ and Pt on PHI that has cyanamide as point defects (Pt–PHI).^14^ Following the procedure in the experimental section, these gC_3_N_4_ samples as supports were all loaded with platinum at 0.2 wt % by reductive deposition from a solution of [(NH_3_)_4_Pt]^2+^. This precursor was selected because a stock solution of this platinum precursor remained stable over time, which we found to enable reproducible platinum loading when preparing the Pt–gC_3_N_4_. As a direct comparison with Pt–gC_3_N_4_, Pt–TiO_2_ prepared in an identical fashion was employed as a benchmark given the well-documented ability of this support material to stabilize metal nanoparticles.^78–80^ Characterization results of these Pt–gC_3_N_4_ samples are presented in Figures S19–S26, which are accompanied by supplementary discussions. These catalysts were tested for the transfer hydrogenation of nitrobenzene into aniline using formic acid as the hydrogen source, a reaction that was selected as a demanding test for these catalytic systems given that NH_3_ and, by extension, amines and therefore aniline can coordinate strongly to Pt. Conversion and product selectivity, as determined by gas chromatography and NMR, are shown in Figure S27–S29, accompanied by supporting discussions. Postcatalytic analyses of Pt–gC_3_N_4_ and Pt–TiO_2_ by XRD and FTIR (blue plots in Figures S19 and S25, respectively) as well as XPS for Pt–Melon-M (Figure S24) showed negligible change compared to their pristine state, from which we inferred that these support materials are structurally stable in the nitrobenzene transfer hydrogenation reaction. In these catalysis experiments, our focus is on the stability of these platinized gC_3_N_4_, which we evaluate in terms of the quantity of platinum remaining on the support and leached into the solution after the transfer hydrogenation, as determined by atomic emission spectroscopy (AES). The procedure is graphically summarized in Figure 5a; Pt quantifications are presented in panels b and c and explained below.

**Figure 5:**
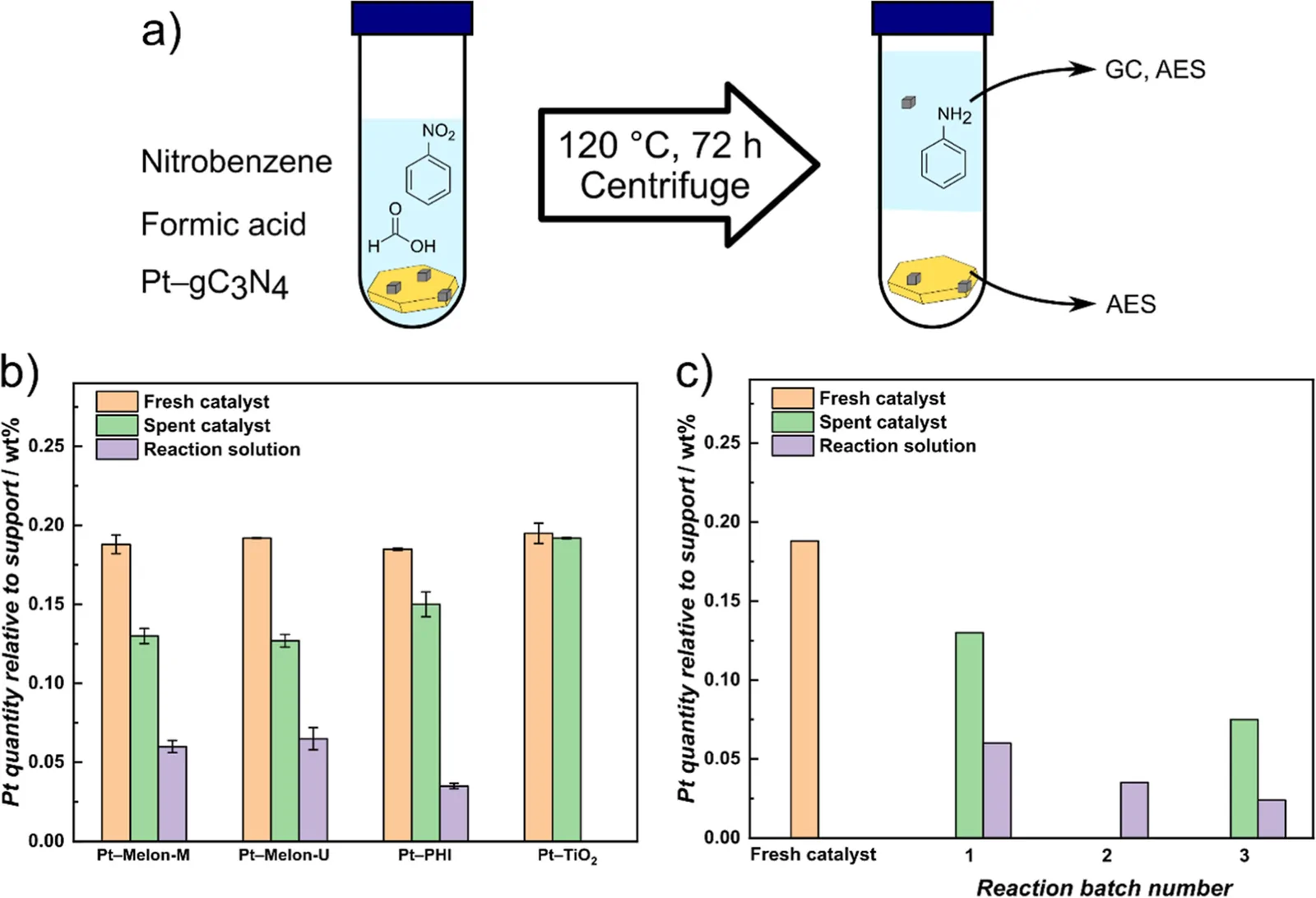
(a) Schematic of the transfer hydrogenation and subsequent centrifugal separation of liquid and solid phases for elemental analyses. (b) Platinum quantity on the solid support before (orange column) and after the transfer hydrogenation (green column) and in the solution (purple column). Average results are reported, with error bars calculated from the standard deviation of three replicates. (c) Platinum quantity remaining on Pt–Melon-M and in the reaction solution as a function of number of catalyst reuses for the transfer hydrogenation. Platinum quantities in b,c are relative to the mass of the support.

The orange columns of Figure 5b show that all freshly prepared catalyst systems have a platinum loading of 0.185–0.195 wt %, close to that of the target of 0.2 wt %. After the transfer hydrogenation, approximately one-third of the platinum was leached from both Pt–Melon-M and Pt–Melon-U, while around one-sixth was leached from PHI, as represented by the green columns. The quantities of platinum leached into the solution after the reaction are represented by the purple columns and are roughly equal to the difference between the fresh and spent catalysts, signifying Pt mass balance in the reaction. Hence, platinum losses from these Pt–gC_3_N_4_ samples are attributed to detachment from support due to the weak coordinative interactions between Pt and gC_3_N_4_, interactions that may have been outcompeted by the coordinating product, aniline. However, this detachment appears to be lessened in PHI, which has the cyanamide groups as defects. In contrast, Pt loading in Pt–TiO_2_ exhibited negligible change, and platinum quantity in the solution was below the detection limit, illustrating that the TiO_2_ support effectively inhibited metal detachment. Notably, high-temperature reductive treatment (200+ °C)^81^ was not required to inhibit Pt leaching from Pt–TiO_2_. Given that the procedure for platinum deposition was identical for Pt–gC_3_N_4_ and Pt–TiO_2_, these results indicate that gC_3_N_4_ may lack the efficacy for stabilizing the platinum catalyst in comparison to TiO_2_ as a classical support material. For the three Pt–gC_3_N_4_ samples, the transfer hydrogenation was also repeated in the absence of the reactant (i.e., heating with stirring for 72 h at 120 °C, the reaction mixture containing only the supported platinum catalyst, water, and formic acid but without nitrobenzene). In these control experiments, platinum leaching into the solution was below the detection limit of the atomic emission spectrometer (Figure S31 for examples of emission spectra), signifying that the dominant cause of platinum leaching is related to the catalytic process, as aggravated by the coordinating product (aniline). We may conclude further that platinum leaching arising from the decomposition of gC_3_N_4_ under the reaction conditions is of a negligible extent, consistent with the chemical stability of this support based on the postcatalytic characterizations by XRD, FTIR, and XPS as mentioned above. To determine whether platinum loss continued for the Pt–gC_3_N_4_, one of the catalysts, Pt–Melon-M, was recovered and reused for a total of three reaction batches. The results in Figure 5c track the platinum quantity in the reaction solution for each batch (purple column) as well as on the Pt–Melon-M after the third reaction (green column). Evidently, platinum continuously leached into the solution in every batch, though in decreasing amounts, which presumably reflects the lower Pt loading at the start of every cycle of reaction. After the third reaction, platinum loading on Pt–Melon-M was below 0.08 wt %, signifying the majority of the original platinum loaded had been lost. Combining these results, the extensive metal leaching observed here is thus consistent with the weak coordinative interactions between Pt and gC_3_N_4_ as inferred from our molecular model studies. At this point, it should be mentioned that previous literature has reported metal–gC_3_N_4_ interactions based on X-ray spectroscopies, electron microscopy, computational modeling, etc.,^11,12,17,82–87^ although postcatalytic analyses confirming metal immobilization and/or the absence of metal leaching are largely absent. The distinction between detecting the presence of such interactions using highly sensitive techniques and confirming their efficacy for catalyst stabilization thus highlights the need to follow best practices in heterogeneous catalysis as published in refs 88–90 to rigorously assess material suitability for supported metal catalysts. Naturally, our results and implications do not necessarily exclude the possibility that specific gC_3_N_4_ formulations could stabilize metallic catalysts, given that stabilizing interactions may depend on gC_3_N_4_ synthesis route (which could affect the type and density of defects) and on the catalyst deposition method. Furthermore, as our catalyst stability tests with nitrobenzene transfer hydrogenation yielded aniline as a coordinating product, these represent a stress test for coordinative stability. In reactions lacking competing ligands, Pt–gC_3_N_4_ may not exhibit the severe metal leaching observed here, making the choice of support materials and/or metal–support combination inherently dependent on the reactions themselves. Along this line, while the scope of this work focuses on platinum catalysts, gC_3_N_4_ may exhibit different abilities to stabilize different metals and oxidation states thereof. Ultimately, achieving the stabilization of metal catalysts (including single-/few-atom catalysts) using the gC_3_N_4_ family of materials as supports will depend on identifying the formulations that exhibit sufficiently robust metal–gC_3_N_4_ interactions for the targeted reactions, an endeavor that can be guided by computational discovery or a better fundamental understanding of heptazine coordination chemistry, alongside rigorous catalyst evaluation.

## Conclusion

Compared to carbon-based supports, the gC_3_N_4_ family of materials is widely considered to be superior for supporting metal catalysts due to their abundant nitrogen-based sites for coordination interaction. To evaluate this premise and to circumvent the structural complexity of gC_3_N_4_ materials, we employed the heptazine molecules melem, cyamelurate, and melonate as model compounds for structurally emulating the terminations and defects of heptazine-based gC_3_N_4_. Focusing on modeling Pt–gC_3_N_4_ as a widely employed catalyst system, we employed these heptazine models to investigate their coordinative interactions with three Pt^2+^ precursors, [(NH_3_)_4_Pt]^2+^, [PtCl_4_]^2–^ and Pt­(NO_3_)_2_. While SC-XRD unambiguously confirmed second-sphere coordination between [(NH_3_)_4_Pt]^2+^ and either cyamelurate or melonate, direct primary-sphere Pt–heptazine complexation was not conclusively observed under the tested conditions regardless of the platinum precursor used. We thus inferred that Pt–heptazine interactions are intrinsically weak, or at least too weak to be detected by the analytical approaches employed here. Translating these findings from molecular models to the heptazine polycondensates, our results further imply that Pt–gC_3_N_4_ interactions may be weaker than expected. This implication was validated by demonstrating extensive metal leaching in Pt–melon and Pt–PHI when employed as catalysts for transfer hydrogenation, particularly in the case here where the reactant (nitrobenzene) is transformed into aniline as a coordinating product. Hence, while gC_3_N_4_ materials have been extolled as excellent catalyst supports (especially for applications in single-/few-atom catalysis), this work highlights the need for rigorous examination of their catalyst–support interactions in immobilizing the catalyst species following best practices.^88,89^ Along this line, assessing the efficacy of catalyst stabilization in other commonly used supports (e.g., carbonaceous materials with/without nitrogen-doping, metal/covalent organic frameworks, etc.^6,91,92^ ) may be likewise warranted, especially if metal leaching is dependent on reaction composition. Alternatively, successful metal immobilization in these other support materials would suggest the presence of alternative stabilizing interactions or that specific synthetic methods impart the desired stability, both of which could reveal approaches to engineering gC_3_N_4_ materials into supports more capable of metal stabilization. In this regard, beyond gC_3_N_4_, the methodology of model compounds presented here could provide the means to investigate coordinative (and potentially other types of) interactions contributing to metal catalyst stabilization across a range of carbonaceous and molecular support materials.

Further afield, since platinum (and other metals) are often loaded as cocatalysts onto gC_3_N_4_ materials in photo­(electro)­catalytic systems, the weaker-than-expected interactions between these two components implied by the results herein could present a limitation to interfacial charge transfer, in turn limiting their overall activity. In finding that cyamelurate and melonate have enhanced hydrogen-bond basicity, these moieties that are associated with defects could play a crucial role in strengthening the interactions between the gC_3_N_4_ materials and either the cocatalyst or the reaction substrate. Further advances in defect engineering in these materials could be a research direction not only for strengthening their ability to stabilize (single-/few-atom) metal catalysts but also accelerating interfacial charge transfer that could raise the (photo)­catalytic reactivity of these catalytic systems.

## Experimental Section

Experimental procedures for the syntheses of key compounds and for catalysis tests are provided in the following. Descriptions of attempted complexation between cyamelurate, melonate, and melem with the three platinum precursors are provided in the Supporting Information, which also contains details of the analytical instruments employed in this work.

### Melon-Melamine (Melon-M)

Melon was synthesized based on published procedures.^13,93^ Melamine (30 g) was weighed in a lidded ceramic crucible, which was then placed in a muffle furnace equipped with an inlet of N_2_ flow set to 5 L min^–1^. The furnace was purged with nitrogen for 2 h with the crucible lid open and then for another 2 h with the crucible lidded. The furnace was then heated under this N_2_ flow to 550 °C at a ramp rate of 5 °C min^–1^ and held for 6 h. After this heating program, the crucible was allowed to cool naturally to room temperature. The resulting yellow solid was ground into a fine powder using a ceramic mortar and pestle, obtaining approximately 15 g of product.

### Melon-Urea (Melon-U)

Urea (85 g) was weighed in a lidded ceramic crucible, which was then placed in a muffle furnace equipped with an inlet of N_2_ flow set to 1 L min^–1^. As for melon-melamine, the furnace was purged, ramped at 5 °C min^–1^ to 550 °C, and held for 6 h under this N_2_ flow. The resulting light yellow solid was ground into a fine powder using a ceramic mortar and pestle, obtaining approximately 4 g of the product.

### Potassium Poly­(heptazine Imide) (PHI)

Based on published procedures,^14,94^ melon-melamine (1 g), potassium thiocyanate (KSCN, 2 g), and KCl (8 g) were weighed out and ground together using a ceramic mortar and pestle for 10 min. The mixture was transferred into an alumina crucible boat, placed in a quartz tube furnace, and purged with N_2_ (100 L h^–1^). Under this flow rate, the mixture was first dehydrated at 120 °C for 2 h, ramped at 10 °C min^–1^ to 400 °C and held for 6 h, then ramped at the same rate to 500 °C and held of 30 min. The dark yellow product was washed with deionized water (5 × 40 mL), then dried in a vacuum oven (70 °C) overnight.

### Potassium Cyamelurate

Following the published procedures,^29^ melon-melamine (10 g) was refluxed with aqueous KOH (100 mL, 2.5 M) for 1 h. The hot mixture was filtered, and the filtrate was allowed to cool before adding ethanol to induce the precipitation of the product, which was isolated by centrifugation (10,000 RCF, 5 min) and dried in vacuum. The product was further recrystallized twice from water by ethanol vapor diffusion to obtain approximately 3.5 g of needlelike crystals.

### Potassium Melonate

Following published procedures,^28^ melon-melamine (3.0 g) and KSCN (6.0 g, predried in 70 °C vacuum oven) were thoroughly mixed in a ceramic mortar and pestle. The mixture was loaded into an alumina crucible boat and placed in a quartz tube furnace, and the tube was purged N_2_ (100 L h^–1^) for 2 h. Afterward, under this same N_2_ flow, the mixture was first dried at 120 °C for 2 h before ramping at 5 °C min^–1^ to 400 °C and held for 1 h, after which the furnace was heated to 500 °C under the same ramp rate and held for 3 h. When the mixture had cooled naturally to room temperature, the resultant yellow solid was stirred with water (40 mL) for 30 min and filtered through Celite, and the product was precipitated from the filtrate by adding excess acetone. The product was recrystallized once more from water by slow evaporation to yield 400 mg of white solid.

### Melem

Following published procedure,^27^ melamine (180 mg) was weighed into a glass tube (inner diameter 12 mm, outer diameter 16 mm) and flame-sealed at a length of 14 cm under N_2_ (1 atm). The tube was placed vertically in a furnace that allowed the top of the tube to stay at ambient temperature, while the bottom of the tube with melamine was heated at a ramp of 1 °C min^–1^ to 450 °C and held for 5 h. After cooling naturally to room temperature, 60–80 mg of the product was recovered as a white solid from the bottom of the tube by carefully breaking it in the middle and avoiding contamination of the sublimate (melamine) from the top of the tube.

### Compound **1**

Potassium cyamelurate (18 mg, 0.035 mmol) was dissolved in deionized water (40 mL). Separately, [(NH_3_)_4_Pt^II^]­(NO_3_)_2_ (20 mg, 0.052 mmol) was also dissolved in deionized water (40 mL). The platinum solution was slowly added to the cyamelurate solution under gentle stirring to ensure complete mixing. 1 mL of this mixture was taken, and crystallization of the product was induced by vapor diffusion of acetonitrile. After several days, a colorless, needle-shaped crystal suitable for single crystal X-ray diffraction (SC-XRD) analysis was obtained. Crystallographic data obtained from SC-XRD analyses can be accessed from the Cambridge Crystallographic Data Centre (CCDC) with the access number 2554749. The contribution of disordered solvent molecules was removed using the SQUEEZE routine in PLATON.^95^ A void of 36 Å^3^ per unit cell was found, containing 17 electrons, which could be H_2_O molecules. Significant residual peaks were located near the platinum atom, which was disordered over three positions (Pt1, Pt1A, and Pt1B) and were refined with a site occupancy ratio of 0.915, 0.041, and 0.039. The structure was refined as 2-component (nonmeroheral) twin using TWIN and BASF instructions in SHELXL. The final twin volume fraction was refined to 0.15.

### Compound **2**

Potassium melonate (34 mg, 0.068 mmol) was dissolved in deionized water (40 mL). Separately, [(NH_3_)_4_Pt^II^]­(NO_3_)_2_ (20 mg, 0.052 mmol) was also dissolved in deionized water (40 mL). The platinum solution was gradually added to the melonate solution under gentle stirring to ensure homogeneous mixing. The resultant mixture was then allowed to stand in the dark at room temperature 1 for week for slow evaporation to dryness, whereupon thin, transparent needle-like crystals formed at the bottom of the beaker, which were subsequently analyzed by single-crystal X-ray diffraction. For other analyses, these crystals were collected by filtration, washed with water, and dried under vacuum at room temperature. Crystallographic data can be accessed from the Cambridge Crystallographic Data Centre (CCDC) with the access number 2554750. The contribution of disordered solvent molecules was removed using the SQUEEZE routine in PLATON. A void of 479 Å^3^ per unit cell was found, containing 181 electrons. When the structure factor data were included for CheckCIF, alerts A and B were generated due to the residual electron density surrounding the two platinum positions. These alerts will be addressed in the section on compound **2** below.

### Preparation of Pt–gC_3_N_4_ Catalysts

Platinum was deposited onto the three gC_3_N_4_ (Melon-M, Melon-U, and PHI) with a targeted loading of 0.2 wt % by a wet chemical method. 200 mg of gC_3_N_4_ was suspended in ethanol, sonicated for 20 min, then stirred for another 10 min. To this suspension was added [(NH_3_)_4_Pt^II^]­(NO_3_)_2_ (0.8 mg from a 9.4 mg mL^–1^ aqueous stock solution for the 0.2 wt % Pt loading), and the resultant mixture suspension was stirred for 1 day. Afterward, a freshly prepared solution of NaBH_4_ (10 μmol or 5× excess, prepared by dissolution at a concentration of ≈2.2 M in 0.1 M NaOH solution) was added and stirred for 2 h. The resultant suspension was isolated by centrifugation, washed with ethanol and water, and then dried at 70 °C in vacuum. Pt–TiO_2_ was prepared identically, swapping out the 200 mg of gC_3_N_4_ for TiO_2_ (AEROXIDE TiO_2_ P25), to provide direct comparison with the Pt–gC_3_N_4_ series.

### Transfer Hydrogenation of Nitrobenzene

This reaction was performed in a thick-walled glass tube of volume 15 mL, which can be sealed with a Teflon screw cap. In a typical reaction, 103 μL (1 mmol) of nitrobenzene was mixed with 152 μL (4 mmol) of formic acid, and the mixture was made up to 5 mL with water. Pt–gC_3_N_4_ or Pt–TiO_2_ (25 mg) was then added, and the tube was sealed and heated at 120 °C with stirring at 1000 min^–1^ for 72 h in an oil bath.

To identify the constituents in the reaction mixture, ^1^H NMR analysis was performed by collecting a sample of the reaction mixture and mixing it with either CDCl_3_ or D_2_O. To quantify the reaction outcome, 25 μL of the solution phase was sampled from the reaction, diluted to 5 mL with 1 mM dodecane as an internal standard in acetonitrile, dried over MgSO_4_, and centrifuged (13,000 RCF, 10 min) prior to analysis by gas chromatography (GC, see instrumentation details in the Supporting Information).

After performing the transfer hydrogenation, the solid catalyst and the reaction solution were separated by centrifugation, and the supernatant was collected for further analysis. The solid precipitate was washed further with ethanol and then dried in 70 °C vacuum oven overnight prior to further analysis. Both solid and liquid phases were subjected to elemental quantification by atomic emission spectroscopy (AES, see Supporting Information for instrumentation details and for the digestion procedure).

## Supplementary Material



## Notes

Details of experimental procedures and analytical instrumentations, additional characterization results of the compounds prepared, and catalytic results of transfer hydrogenation, all of which are accompanied by detailed discussions (DOCX)
