# Supplementary material for: Molecular Heptazine–Platinum Complexes to Model Metal–Support Coordination in Platinized Graphitic Carbon Nitride: Insights into Catalyst Stability and Support Suitability
Source: Inorg Chem. 2026 Sep 17;65(39):23345–58. doi: 10.1021/acs.inorgchem.6c04290 (PMC13644308; doi:10.1021/acs.inorgchem.6c04290)
Supplement: Supplementary file 1 [file ic6c04290_si_001.docx]

Supporting Information

**Molecular heptazine–platinum complexes to model metal-support coordination in platinized graphitic carbon nitride: insights into catalyst stability and support suitability**

Chi-Feng Lu,† Tsu-Lien Hung,‡ Jiliang Zhang,§ Vincent Wing-hei Lau†*

†Department of Chemistry, National Cheng Kung University, No. 1 University Road, Tainan City 701, Taiwan (ROC)

‡Core Facility Center, National Cheng Kung University, No. 1 University Road, Tainan City 701, Taiwan (ROC)

§School of Materials Science and Engineering, Dalian Jiaotong University, Dalian 116028, P. R. China

*Email: vincentwhlau@gs.ncku.edu.tw

Table of Contents

[Experimental details	3](#_Toc229926589)

[Instrumental details	10](#_Toc229926590)

[Characterization of the three platinum precursors	14](#_Toc229926591)

[Characterization of compound **1**	19](#_Toc229926592)

[Characterization of solid residues of potassium cyamelurate and K_2_PtCl_4_ & Pt(NO_3_)_2_	23](#_Toc229926593)

[UV-Vis titration of K_2_PtCl_4_ and Pt(NO_3_)_2_ with potassium cyamelurate	29](#_Toc229926594)

[Characterization of compound **2**	32](#_Toc229926595)

[Characterization of solid residues of potassium melonate and K_2_PtCl_4_ & Pt(NO_3_)_2_	38](#_Toc229926596)

[UV-Vis titration of K_2_PtCl_4_ and Pt(NO_3_)_2_ with potassium melonate	44](#_Toc229926597)

[Product characterization from attempted complexation with melem in aqueous suspension	47](#_Toc229926598)

[Product characterization from attempted complexation with melem in DMSO solution	53](#_Toc229926599)

[Characterization of Pt–gC_3_N_4_	59](#_Toc229926600)

[Characterization of Pt–TiO_2_ catalysts	67](#_Toc229926601)

[Transfer hydrogenation of nitrobenzene into aniline as catalyzed by Pt–gC_3_N_4_ & Pt–TiO_2_	72](#_Toc229926602)

# Experimental details

**Attempted complexation between cyamelurate and tetrachloroplatinate**: Potassium cyamelurate (20 mg, 0.06 mmol) was dissolved in deionized water (10 mL). Separately, K_2_PtCl_4_ (17 mg, 0.04 mmol) was also dissolved in deionized water (10 mL). The platinum solution was slowly added to the cyamelurate solution under gentle stirring to ensure complete mixing; no color change was observed at this point. 3 mL of this mixture was taken and allowed to evaporate slowly to dryness at ambient conditions in a beaker, yielding a purple powder at the bottom.

**Attempted complexation between melonate and tetrachloroplatinate**: Potassium melonate (21 mg, 0.042 mmol) was dissolved in deionized water (10 mL). Separately, K₂PtCl₄ (12 mg, 0.029 mmol) was also dissolved in deionized water (10 mL). The platinum solution was slowly added to the melonate solution under gentle stirring to ensure complete mixing; no color change was observed. 3 mL of this mixture solution was allowed to evaporate slowly at ambient conditions in a beaker, yielding a yellow powder at the bottom.

**Attempted complexation between cyamelurate and platinum nitrate**: Potassium cyamelurate (20 mg, 0.06 mmol) was dissolved in 10 mL of deionized water. Separately, Pt(NO_3_)_2_ (12.76 mg, 0.04 mmol) was also dissolved in deionized water (10 mL). The two solutions were slowly mixed together; no color change was observed. 3 mL of this mixture solution was taken for slow evaporation to dryness at ambient conditions in a beaker, yielding a yellow powder at the bottom.

**Attempted complexation between melonate and platinum nitrate**: Potassium melonate (21 mg, 0.042 mmol) was dissolved in 10 mL of deionized water. Separately, Pt(NO_3_)_2_ (8.9 mg, 0.028 mmol) was dissolved in deionized water (10 mL). The two solutions were slowly mixed together; no color change was observed. 3mL of this mixture solution was taken for slow evaporation to dryness at ambient conditions in a beaker, yielding a yellow powder at the bottom.

**Attempted complexation between melem and tetraammineplatinum (method 1)**: Melem (10 mg, 0.046 mmol) was suspended in deionized water (10 mL). Separately, a solution of [(NH_3_)_4_Pt^II^](NO_3_)_2_ (17.8 mg, 0.046 mmol) in water (10 mL) was prepared, and then added to the melem suspension and stirred for 1 day. The residual solid was isolated by centrifugation (13000 RCF, 8 min), washed with water twice, and then dried at 70°C in vacuum overnight. Both supernatant and the solid residue were subsequently analyzed.

**Attempted complexation between melem and tetraammineplatinum (method 2)**: Melem (10 mg, 0.046 mmol) was dissolved in dimethyl sulfoxide (DMSO, 10 mL). Separately, a 10 mL DMSO solution containing [(NH_3_)_4_Pt^II^](NO_3_)_2_ (17.8 mg, 0.046 mmol) was prepared and added to the melem-DMSO solution. The DMSO solution mixture was left to stand at ambient conditions for crystallization in the dark. After three weeks, the solution turned cloudy indicative of the formation of a precipitate, which was isolated by centrifugation (13000 RCF, 8 min), washed with water twice, and then dried at 70°C in vacuum overnight prior to further analysis.

**Attempted complexation between melem and tetrachloroplatinate**: Melem (10 mg, 0.046 mmol) was suspended in deionized water (10 mL). Separately, a solution of K_2_PtCl_4_ (14.67 mg, 0.046 mmol) in deionized water (10 mL) was prepared, added to the melem suspension, and stirred for 1 day. Afterward, the suspended solid was isolated by centrifugation (13000 RCF, 8 min), washed twice with water, then dried at 70°C in vacuum overnight prior to further analysis.

**Attempted complexation between melem and platinum nitrate**: Melem (10 mg, 0.046 mmol) was suspended in deionized water (10 mL). Separately, a solution of Pt(NO3)2 (19.1 mg, 0.046 mmol) in deionized water (10 mL) was prepared, added to the melem suspension, and stirred for 1 day. Afterward, the suspended solid was isolated by centrifugation (13000 RCF, 8 min), washed twice with water, then dried at 70°C in vacuum overnight prior to further analysis.

# Instrumental details

**Fourier-transform infrared spectroscopy (FTIR)**: FTIR spectra were recorded in attenuated total reflection (ATR) mode using a PerkinElmer Frontier MIR Spectrometer. The spectrometer had a MIRacle Single Reflection ATR accessory (Pike) with a diamond window. Each sample was scanned four times in the range of 4000–400 cm⁻¹ with a resolution of 1 cm⁻¹.

**Powder X-ray diffraction pattern (PXRD)**: PXRD patterns were recorded on a Bruker D8-Advance X-ray diffractometer using Cu Kα radiation (λ = 1.5418 Å). The instrument operated at a voltage of 40 kV and a current of 25 mA. Typically, the sample was flattened on a zero-background silicon wafer sample holder supplied by the manufacturer. Samples that are air-/moisture-sensitive or hygroscopic/deliquescent are handled inside a glove box, where the powdered sample was deposited onto Bruker’s polymer sample holder with a shallow well and then sealed with Kapton tape, which inevitably leads to a large background signal. All patterns were collected in the range of 5–60° 2θ with 1000 steps over 120 seconds. For comparison, calculated powder XRD patterns were generated using Mercury software 4.2.0 from the compound’s published Crystallographic Information File (CIF).

**Solution state Nuclear Magnetic Resonance (NMR)**: Solution-state ^195^Pt and ^13^C NMR spectra were recorded using a Bruker AV-500 spectrometer.

**X-ray photoelectron spectroscopy (XPS)**: XPS spectra were collected using a VersaProbe 4 spectrometer (PHI) with Al Kα radiation (1486 eV). The sample mount was a piece of glass slide cut to 1×1 cm, which was sonicated in ethanol for 5 min and blow-dried with N_2_ before use. Then, a non-conductive double-sided tape was attached to the glass slide, and the powdered sample was dusted onto the tape until the entire tape was covered with the sample. After blowing off excess sample, the glass slide was attached to the spectrometer’s sample holder using adhesive tape. All XPS data were analyzed using XPSPEAK4.1 software. Shirley background was used for all spectra, which were fitted using the least-squares method. For spectral fittings where applicable, the following constraints were applied for the spin-orbital coupling energy: 3.4 eV for Pt4f spectra, 1.6 eV for Cl2p, and 2.8 eV for K2p. In all XPS spectra, raw data points are plotted as black scatter plot, the fitted peaks as colored line plots, the peak sum as red plot, Shirley background as dashed gray line, and residual as magenta line plot beneath each spectrum.

**Elemental Analysis (EA)**: An elemental analyzer (UNICUBE) was used for CNH quantification by the combustion method. Each sample of amount 1–2 mg was analyzed in duplicate, and the average results were reported. Instrument calibration was performed using sulfanilic acid.

**Microwave Plasma Atomic Emission Spectroscopy (MP-AES)**: An Agilent MP-AES 4210 spectrometer was used for the analyses of the inorganic element, in this case, platinum with an emission wavelength of 265.945 nm. Calibration standard solutions were prepared by diluting commercially available stock solution (1000 ppm) to concentrations of 10, 5, 2.5 and 1 ppm. HNO_3_ (0.3 N) was used as diluent for preparing both calibration standards or analyte solutions and as carrier solution for the spectrometer injection system. Carryover was prevented by setting uptake time to 70 s with a 15 s stabilization time, the time between uptake and spectrum collection that allows for plasma stabilization. After each analysis, this nitric acid solution was used to rinse the injection system for 60 s. For MP-AES analysis of platinum in solid samples, digestion was performed using aqua regia as follows. The sample (around 25 mg) was accurately weighed out in an autoclave with a Teflon liner, to which a mixture of HCl (3 mL, 37%) and HNO_3_ (1 mL, 70%) was added. The autoclave was sealed, heated at 80 °C for 2 h, and the resultant solution was appropriately diluted with the aforementioned HNO_3_ solution for MP-AES analysis. For MP-AES analysis of liquid samples, the samples were first evaporated to complete dryness and the residue was digested with aqua regia in an identical fashion as for solid samples described above.

**Single crystal X-ray diffraction (SCXRD)**: The diffractometer was a Bruker D8 VENTURE Four Circle Kappa with a microfocus sealed tube using a multilayer mirror as monochromator and a Bruker PHOTON III CPAD detector. The diffractometer is equipped with an Oxford Cryostream 700 low temperature device and uses Mo *K_α_* radiation (λ = 0.71073 Å). All data were integrated with SAINT V8.42 (Bruker AXS SE). A multi-scan absorption correcting using TWINABS Bruker was applied.^1^ Structure solutions were obtained by Intrinsic Phasing methods with SHELXT 2018/2 and refined by full-matrix least-squares methods against *F*^2^ using SHELXL-2019/2.^2, 3^ All non-hydrogen atoms were refined with anisotropic displacement parameters. The heteroatom-bound hydrogen atoms were refined isotropically on calculated positions using a riding model with their *U*_iso_ values constrained to 1.5 times the *U*_eq_ of their pivot atoms for terminal sp^3^ carbon atoms and 1.2 times for all other carbon atoms.

**UV-Vis spectroscopy**: UV-Vis spectra were recorded on a Thermo Fisher Scientific Evolution One Plus spectrometer. Samples were solubilized in a quartz cuvette (1 cm pathlength) and the spectra were collected in transmission mode in the wavelength range 190–1100 nm with a resolution of 1 nm. Pure solvents were used for blank correction.

**Gas Chromatography with Flame Ionized detector (GC-FID)**: GC-FID was performed on a Shimadzu GC-2014 gas chromatography system equipped with a capillary column (SGE Analytical Science, BP5 (methylpolysiloxane with 5% phenyl, 1 µm), 30 m × 0.32 mm). 1 µL of the sample was taken up using a needle and syringe (SGE Analytical Science, 10 µL) and injected through the septum into the column, which has a heating program of 90 °C held constant for 3 min, then ramping to 210°C at a rate of 20 °C min^-1^.

**High-Angle Annular Dark-Field Scanning Transmission Electron Microscopy (HAADF-STEM):** Electron microscope images were collected on a JEOL JEM-2100F CS HAADF-STEM operating at 200 kV and 131 µA. Samples were prepared as follows. About 4 mg of the solid was sonicated for 45 min in 4 mL water to yield the sample suspension. 10 μL of this suspension was then deposited onto a lacey carbon support film or carbon-coated copper mesh, and then dried in vacuum at 70 °C for 3 d prior to STEM analysis.

# Characterization of the three platinum precursors

Coordination of each platinum precursor to the heptazine-based molecules is anticipated to induce shifts in the wavenumbers of the FTIR bands as well as the BEs of elemental environments in XPS, in addition to forming new crystallographic phase(s) with distinct diffraction patterns. Hence, to confirm successful coordination between the heptazine ligand and the platinum metal center, the three platinum precursors were first characterized: the FTIR spectra and XRD patterns are plotted in Figure S1, while the XPS spectra are shown in Figure S2. Note that, since all three platinum precursors are hygroscopic, XRD patterns were collected by sealing the powdered sample in Kapton tape that inevitably introduced the broad peak observed at around 18° 2θ.

Figure S1 Characterization results of the three platinum precursors by a) FTIR and b) XRD.

Figure S2 XPS spectra of [(NH_3_)_4_Pt^II^](NO_3_)_2_ (top), K_2_PtCl_4_ (middle) and Pt(NO_3_)_2_ (bottom); fitting parameters are tabulated in Table S1.

Table S1 Peak fitting parameters of the spectra in Figure S2 of [(NH_3_)_4_Pt^II^](NO_3_)_2_, K_2_PtCl_4_ and Pt(NO_3_)_2_. Spin-orbital coupling constant was constrained to 1.6 eV for the Cl2p peak and to 2.8 eV for the K2p during fitting.

| Element & peak number | BE (eV) | FWHM | Integral | χ^2^ |
| --- | --- | --- | --- | --- |
| **[(NH_3_)_4_Pt^II^](NO_3_)_2_** |  |  |  |  |
| Pt4f7/2 – peak 1 | 73.2 | 1.84 | 7752 | 7.75 |
| N1s – peak 1 | 400.1 | 1.79 | 2103 | 0.26 |
| N1s – peak 2 | 406.6 | 1.58 | 884 |  |
| O1s – peak 1 | 532.2 | 2.00 | 9510 | 9.26 |
|  |  |  |  |  |
| **K_2_PtCl_4_** |  |  |  |  |
| Pt4f7/2 – peak 1 | 72.6 | 1.19 | 10288 | 8.47 |
| Cl2p3/2 – peak 1 | 198.6 | 1.12 | 6612 | 1.79 |
| K2p3/2 – peak 1 | 292.7 | 1.17 | 5082 | 1.00 |
| C1s – peak 1 | 284.8 | 1.39 | 32909 | 2.28 |
| C1s – peak 2 | 286.4 | 1.31 | 7409 |  |
| C1s – peak 3 | 288.7 | 1.07 | 4046 |  |
|  |  |  |  |  |
| **Pt(NO_3_)_2_** |  |  |  |  |
| Pt4f7/2 – peak 1 | 73.3 | 1.50 | 1574 | 2.09 |
| Pt4f7/2 – peak 2 | 75.4 | 1.57 | 14495 |  |
| O1s – peak 1 | 532.4 | 2.37 | 9226 | 1.03 |
| C1s – peak 1 | 284.8 | 1.77 | 5484 | 0.15 |
| C1s – peak 2 | 286.5 | 1.41 | 644 |  |
| C1s – peak 3 | 288.8 | 1.8 | 549 |  |

In the discussion below of the XPS spectra, our results are compared with literature findings. For example, BE of platinum species can be compared to the results of reference 4 and 5, oxygen species from water to reference 6, and nitrogen and oxygen species of nitrate to reference 7. This reference 7 also describes radiolytic decomposition of nitrate under x-ray irradiation within an XPS spectrometer. For tetraammineplatinum(II) nitrate, the BE of the Pt4f7/2 peak at around 73 eV is consistent with its oxidation state of Pt^2+^, while one of two N1s peaks at BE ≈400 eV can be assigned to NH_3_, as consistent with literature.^5^ The other N1s peak at BE 406.6 eV may be nitrate or its radiolytic decomposition product(s), one example being NO_2_^–^ from reference 7. Differentiating nitrate and nitrite by their O1s peaks (respectively, BE of 533.0 and 531.5 eV also from reference 7) is difficult given that this signal is broad and encompasses the BE value range of adsorbed water, noting that this platinum salt is hygroscopic.

For potassium tetrachloroplatinate, due to the ease of chloride reduction during XPS analysis, the narrow scan for this element was collected first prior to performing the survey scan and narrow scans for the remaining elements. The BE for the chloride is around 0.4 eV lower than that of reference 5 (BE 198.8 eV after correcting for calibration), which may suggest that the sample had partially reduced during the analysis. In turn, platinum appears to also have been partially reduced, as our value for the BE of Pt is around 0.6 eV lower than that of reference 5 (BE 73.0 eV after correcting for calibration), although still within the range consistent with its oxidation state of Pt^2+^. Note that the fitting results of the C1s signals in the range 282–290 eV are from the double-sided adhesive tape used for mounting the powder, and are identical to those presented in our previous publication.^8^

For Pt(NO_3_)_2_, two platinum environments can be observed with BE of 73.3 eV and 75.4 eV, which are respectively assigned to Pt^2+^ and Pt^4+^, the latter possibly due to oxidative radiolytic decomposition. Similar oxidation of Pt^2+^ species to Pt^4+^ due to long exposure to x-ray in XPS has been observed.^9^ Radiolytic oxidation of Pt^2+^ could be accompanied by reduction of the nitrate to volatile species, which has also been observed during XPS analysis.^7^ This could explain the poor signal intensity of the nitrate XPS signal, which was not fitted due to low signal-to-noise. Nevertheless, two peaks above the noisy baseline at around 405 and 407 eV are discernible, and may be assigned to nitrate and nitrite, respectively.^5, 7^ As for the O1s spectrum, similar to the cases above, the O1s peak is broad with BE in the range expected for NOx and for adsorbed water, the latter of which could be present in substantial quantity considering that Pt(NO_3_)_2_ is very hygroscopic. Note that all fitting results for the C1s region are identical (within the spectrometer’s energy resolution) to those of Table S1, and correspond to the double-sided tape used for mounting powdered samples.

# Characterization of compound **1**

Table S2 Crystal data and structural refinements for compound **1**.

| Empirical formula | C6 H12 N11 O3 Pt1.03 |
| --- | --- |
| Formula weight | 486.4 |
| Temperature [K] | 100(2) |
| Crystal system | monoclinic |
| Space group (number) | $P2_{1}/m$ (11) |
| *a* [Å] | 3.6702(19) |
| *b* [Å] | 13.669(7) |
| *c* [Å] | 12.638(7) |
| α [°] | 90 |
| β [°] | 94.845(7) |
| γ [°] | 90 |
| Volume [Å^3^] | 631.8(6) |
| *Z* | 2 |
| *ρ*_calc_ [g cm^−3^] | 2.557 |
| *μ* [mm^−1^] | 11.425 |
| *F*(000) | 458 |
| Crystal size [mm^3^] | 0.07×0.11×0.15 |
| Crystal colour | Colourless |
| Crystal shape | Block |
| Radiation | Mo *K_α_* (λ=0.71073 Å) |
| 2θ range [°] | 4.40 to 52.00 |
| Index ranges | −4 ≤ h ≤ 4 −16 ≤ k ≤ 16 −15 ≤ l ≤ 15 |
| Reflections collected | 24450 |
| Independent reflections | 2479  *R*_int_ = 0.0415 *R*_sigma_ = 0.0750 |
| Completeness to θ = 25.242° | 99.6 |
| Data / Restraints / Parameters | 2479 / 43 / 113 |
| Goodness-of-fit on *F*^2^ | 1.117 |
| Final *R* indexes [*I*≥2σ(*I*)] | *R*_1_ = 0.0770 w*R*_2_ = 0.1917 |
| Final *R* indexes [all data] | *R*_1_ = 0.0884 w*R*_2_ = 0.2057 |
| Largest peak/hole [eÅ^−3^] | 3.53/−4.00 |

Table S3 Atomic coordinates, equivalent displacement parameters (Å^2^) and occupancies for compound **1**

| Atom | Site | x | y | z | Ueq | Occ |
| --- | --- | --- | --- | --- | --- | --- |
| Pt1 | 2e | 0.5633(3) | 0.75 | 0.16168(10) | 0.0349(5) | 0.915(16) |
| Pt1A | 2e | 0.594(6) | 0.75 | 0.225(3) | 0.0349(5) | 0.041(3) |
| Pt1B | 2e | 0.530(7) | 0.75 | 0.096(3) | 0.0349(5) | 0.039(3) |
| O2 | 4f | 0.044(4) | 0.4981(10) | 0.1505(10) | 0.051(3) | 1 |
| O1 | 2e | 0.888(5) | 0.25 | 0.5682(13) | 0.041(4) | 1 |
| N1 | 4f | 0.625(6) | 0.8552(12) | 0.2739(15) | 0.058(5) | 1 |
| N2 | 4f | 0.614(5) | 0.3370(11) | 0.4312(12) | 0.051(4) | 1 |
| N3 | 4f | 0.495(5) | 0.6429(11) | 0.0505(13) | 0.050(4) | 1 |
| N4 | 4f | 0.330(5) | 0.4217(11) | 0.2935(13) | 0.048(4) | 1 |
| N5 | 2e | 0.321(6) | 0.25 | 0.2880(17) | 0.044(5) | 1 |
| N6 | 4f | 0.024(4) | 0.3344(10) | 0.1458(12) | 0.041(3) | 1 |
| C1 | 2e | 0.124(7) | 0.25 | 0.191(2) | 0.041(4) | 1 |
| C2 | 2e | 0.715(7) | 0.25 | 0.481(2) | 0.044(4) | 1 |
| C3 | 4f | 0.121(5) | 0.4196(13) | 0.1953(15) | 0.045(3) | 1 |
| C4 | 4f | 0.429(5) | 0.3401(15) | 0.3362(14) | 0.046(3) | 1 |

Table S4 CHN elemental analyses of compound **1** as mass percentage and as molar ratio.

| Element | Mass percentage | Molar ratio | Molar ratio (normalized to 6 C) |
| --- | --- | --- | --- |
| C | 14.76 | 1.229 | 6 |
| N | 30.36 | 2.167 | 11 |
| H | 3.022 | 3.022 | 15 |

Figure S3 XPS spectra of compound **1** in the C1s, N1s, O1s and Pt4f regions; fitting parameters are tabulated in Table S5

Table S5 Peak fitting parameters of the spectra (Figure S3) of compound **1**

| Element & peak number | BE (eV) | FWHM | Integral | χ^2^ |
| --- | --- | --- | --- | --- |
| C1s – peak 1 | 284.7 | 2.31 | 1536 | 1.47 |
| C1s – peak 2 | 288.0 | 1.99 | 845 |  |
| N1s – peak 1 | 398.2 | 2.48 | 2185 | 0.13 |
| N1s – peak 2 | 400.0 | 2.22 | 1155 |  |
| O1s – peak 1 | 530.6 | 2.48 | 1522 | 1.26 |
| Pt4f – peak 1 | 73.0 | 2.35 | 5280 | 2.80 |

Figure S4 FTIR spectrum of compound **1** compared with spectra of potassium cyamelurate, cyameluratic acid, and the platinum precursor [(NH_3_)_4_Pt^II^](NO_3_)_2_. Shown on the right are the principal tautomer of cyamelurate anion as a metal salt and as an acid.

# Characterization of solid residues of potassium cyamelurate and K_2_PtCl_4_ & Pt(NO_3_)_2_

As stated in the main text, mixing a solution of potassium cyamelurate with either aqueous K_2_PtCl_4_ or Pt(NO_3_)_2_ did not yield a precipitate, from which we inferred that an insoluble coordination polymer did not form that would provide evidence for Pt–cyamelurate complexation. To verify this inference more thoroughly, the two aqueous mixtures of potassium cyamelurate with either of the two platinum precursors were evaporated to dryness and the residues were examined by XRD and FTIR, and the results are shown in Figure S5.

Figure S5 Characterization by a) XRD and b) FTIR of the solid residues obtained by mixing aqueous solution of potassium cyamelurate and of K_2_PtCl_4_ or Pt(NO_3_)_2_, followed by evaporation to dryness.

The XRD patterns are largely amorphous, although some unidentified phases were detected in the residue of Pt(NO_3_)_2_ and cyamelurate. These phases are likely related to oxides of platinum (e.g. PtO or PtO_2_) since Pt(NO_3_)_2_ is susceptible to hydrolysis.^10^ Considering that the reflection at 27.33° 2θ was also observed in the solid residue from mixing Pt(NO_3_)_2_ and potassium melonate as described in the next section, the crystalline phase is unlikely to originate from Pt–cyamelurate complexation. Hence, to detect coordinative bonds between platinum and the cyamelurate, we examined the FTIR spectra, focusing on the bands related to heptazine vibrations and whether there is any wavenumber shift before and after platinum complexation. Table S6 tabulates the wavenumbers of the heptazine vibrations in the range of 1476–1595 cm^-1^ and the heptazine out-of-plane vibration mode at 776–812 cm^-1^ for the two solid residues (entry 4 and 5) and compare them against cyamelurate (tri-potassium salt and the acidic form) as well as compound **1**.

Table S6 Wavenumbers in cm^-1^ for the IR bands corresponding to the heptazine vibration mode and the heptazine out-of-plane vibration for compound **1** and the two other attempts at Pt–cyamelurate complexation, compared with those for potassium cyamelurate and cyameluric acid.

| Entry | Sample | Heptazine vibrational | Heptazine out-of-plane vibration |
| --- | --- | --- | --- |
| 1 | Potassium cyamelurate | 1476 | 812 |
| 2 | Cyameluric acid | 1595 | 776 |
| 3 | Compound **1** | 1524 | 800 |
| 4 | Potassium cyamelurate + K_2_PtCl_4_ | 1506 | 806 |
| 5 | Potassium cyamelurate + Pt(NO_3_)_2_ | 1542 | 793 |

As seen in this table, for the two residues, the wavenumbers of their bands are similar to those of compound **1**, which does not have first-sphere platinum–cyamelurate coordination. Moreover, their wavenumbers lie between those of potassium cyamelurate and cyameluric acid, which could suggest that the wavenumber shifts could be attributed to protonation of this heptazine molecule, rather than from complexation. Since results from FTIR are inconclusive as to whether Pt–cyamelurate coordination occurred using these two platinum precursors, these residues were also analyzed by XPS, where we anticipate BE shifts upon changes to the coordination environment. Figure S6 and Figure S7 show the spectra of the residue from K_2_PtCl_4_ and Pt(NO_3_)_2_, respectively, while the peak fitting parameters are tabulated in Table S7.

Figure S6 XPS spectra of the solid residue obtained by evaporating an aqueous solution of potassium cyamelurate and K_2_PtCl_4_; fitting parameters are tabulated in Table S7.

Figure S7 XPS spectra of the solid residue obtained by evaporating an aqueous solution of potassium cyamelurate and Pt(NO_3_)_2_; fitting parameters are tabulated in Table S7.

Table S7 Fitting parameters of the spectra in Figure S6 and Figure S7.

| Element & peak number | BE (eV) | FWHM | Integral | χ^2^ |
| --- | --- | --- | --- | --- |
| **K_2_PtCl_4_ + potassium cyamelurate** | |  |  |  |
| C1s – peak 1 | 284.8 | 1.49 | 12104 | 1.04 |
| C1s – peak 2 | 288.5 | 1.25 | 3883 |  |
| N1s – peak 1 | 399.1 | 2.08 | 7311 | 1.97 |
| N1s – peak 2 | 400.9 | 2.51 | 1548 |  |
| O1s – peak 1 | 532.0 | 2.43 | 21639 | 4.81 |
| Pt4f7/2 – peak 1 | 72.8 | 1.46 | 25429 | 14.6 |
| K2p3/2 – peak 1 | 292.9 | 1.34 | 48766 | 3.77 |
| Cl2p3/2 – peak 1 | 198.2 | 1.19 | 25828 | 5.76 |
| **Pt(NO_3_)_2_ + potassium cyamelurate** | |  |  |  |
| C1s – peak 1 | 284.8 | 1.77 | 5484 | 0.15 |
| C1s – peak 2 | 286.5 | 1.41 | 644 |  |
| C1s – peak 3 | 288.8 | 1.8 | 549 |  |
| N1s – peak 1 | 398.1 | 1.41 | 6364 | 0.74 |
| N1s – peak 2 | 400.1 | 2.09 | 1836 |  |
| N1s – peak 3 | 403.9 | 1.65 | 1383 |  |
| N1s – peak 4 | 406.9 | 1.32 | 2298 |  |
| O1s – peak 1 | 530.8 | 1.99 | 20432 | 2.01 |
| O1s – peak 2 | 532.4 | 1.63 | 16773 |  |
| Pt4f7/2 – peak 1 | 74.4 | 1.33 | 26071 | 12.1 |

We first discuss the XPS spectra of the residue from K_2_PtCl_4_ and cyamelurate. When compared with K_2_PtCl_4_ (Figure S2), the Pt4f7/2 and K2p3/2 peaks have BE values differing by less than 0.2 eV, which is insignificant given that this is the energy resolution of the XPS spectrometer. The Cl2p3/2 peak shifted to lower BE by 0.4 eV, which is within the instrument’s spectral resolution. While this shift might be real and could arise from Pt–cyamelurate complexation, one alternative explanation for Cl2p3/2 peak shift could be related to changes in the second-sphere coordination, while first-sphere coordination remains unchanged (i.e. platinum staying as [PtCl_4_]^2-^). Considering this ambiguity, we examined the N1s and O1s signals that originate from the cyamelurate. When compared with cyameluric acid from our previous publication (N1s peaks at 399.1 and 400.5 eV),^8^ the BE values of the two N1s peaks differ by less than 0.4 eV, again within the spectral resolution. As for the O1s peak, it is too broad for meaningful interpretation, although we note that the BE value of the peak center lies between those of cyameluric acid and potassium cyamelurate. Hence, similar to the issues in the FTIR results, while the minor BE shifts in the XPS could suggest changes in coordination environment, we cannot rule out that these peak shifts (if indeed real) originate from partial protonation of the cyamelurate anion, as in the case of compound **1**, rendering ambiguous any evidence supporting the Pt–cyamelurate complexation through displacement of the chloro ligand in K_2_PtCl_4_ by cyamelurate.

As for the spectra of the residue from the mixture of potassium cyamelurate and Pt(NO_3_)_2_, the Pt4f7/2 signal of the residue has BE 1 eV lower than that of the main peak of the Pt precursor, which could indicate a change of coordination surrounding the Pt center. While this could evidence the formation of Pt–cyamelurate, an alternative explanation is that this shift originated from Pt(NO_3_)_2_ hydrolysis to form PtO or PtO_2_.^10^ Indeed, the BE of the Pt4f7/2 peak of the residue at 74.4 eV matches that of PtO_2_ in reference ^11^. As for the N1s and O1s signals, their BE values differ by less than 0.4 eV when compared to those of potassium cyamelurate (398.1 and 400.5 eV from our previous publication^8^) and of Pt(NO_3_)_2_ (Figure S2), a shift that is within the spectral resolution of XPS spectroscopy as mentioned above. As in the case of K_2_PtCl_4_, while these peak shifts might be real, it is ambiguous as to whether they are related to Pt–cyamelurate complexation, given that we cannot rule out partial hydrolysis to PtO_(2)_ to explain the change in coordination environment.

# UV-Vis titration of K_2_PtCl_4_ and Pt(NO_3_)_2_ with potassium cyamelurate

Considering the ambiguity in analyzing the solid residues as to whether cyamelurate displaced the chloro/nitrato ligand of K_2_PtCl_4_ and Pt(NO_3_)_2_, we next attempted to detect Pt–cyamelurate coordination in the solution phase by performing UV-Vis titration experiments, the results of which are shown in Figure S8.

Figure S8 UV-Vis titration of potassium cyamelurate solutions with K_2_PtCl_4_ (a-b) and with Pt(NO_3_)_2_ (c).

Figure S8a shows the titration of K_2_PtCl_4_ (1 mL, 5.1 mM, dark gray plot) with potassium cyamelurate solution (6.1 mM, orange plot), whereby mixtures 1–8 in the legend refer to increasing addition of the cyamelurate solution in 140 μL aliquots up to a total of 8×140 μL = 1120 μL for mixture 8 (yielding a molar ratio of 4 cyamelurate to 3 Pt^2+^). Other than a change in band intensity due to dilution with the titrant, no wavelength shift is observed for the three absorption bands of K_2_PtCl_4_ at 327, 390 and 473 nm, as indicated by the hairlines drawn, suggesting that the platinum center remained as [PtCl_4_]^2-^. To determine whether the cyamelurate ligand exhibited shifts in its UV-Vis absorption band at 273 nm, this titration was repeated but at a lower concentration such that this cyamelurate band is observable as in Figure S8b. Note that we focus on this band at 273 nm rather than the band at 212 nm, given that the latter overlaps with that of K_2_PtCl_4_. Here, K_2_PtCl_4_ (1 mL, 204 μM, dark gray plot) was titrated with potassium cyamelurate solution (240 μM, tan plot), whereby mixtures 1–7 in the legend refer to increasing addition of the cyamelurate solution in 140 μL aliquots up to a total of 7×140 μL = 980 μL for mixture 7 (final molar amount of 204 nmol of K_2_PtCl_4_ and 235 nmol of cyamelurate). With increasing titrant amount, the intensity of cyamelurate UV band increases, although no wavelength shift is discernible as indicated by the hairline drawn. This results likewise suggests negligible change in the electronic properties of cyamelurate, in turn indicating no donation of electron densities as would be expected of coordination.

Figure S8c shows the titration of Pt(NO_3_)_2_ (1 mL, 6.96 μM, gray plot) with potassium cyamelurate solution (24 μM, orange plot), whereby mixtures 1–8 in the legend refer to increasing addition of the cyamelurate solution in 48 μL aliquots up to a total of 8×48 μL = 384 μL for mixture 8 (final molar amount of 7.0 nmol of Pt(NO_3_)_2_ and 9.2 nmol of cyamelurate). The spectrum of platinum nitrate only shows a broad absorption from 200 nm stretching into the visible region until 450 nm. While this is not amendable to peak-shift analyses, it does not overlap with the bands of cyamelurate, such that we could monitor its band at 212 nm for any peak shift. As observed in these spectra, the intensity of this cyamelurate band increases in its intensity with the addition of the cyamelurate titrant solution. However, no wavelength change is observed band as indicated by the hairline, again suggesting negligible change to the electronic properties of this heptazine compound that would otherwise indicate coordination based on the donation of electron densities. These results from solid-state and solution-state analyses of the mixtures of cyamelurate and the two platinum precursors were ambiguous as to whether Pt–cyamelurate complexation occurred, leading to the conclusion that such coordinative bond is (negligibly) weak if present at all. In turn, these results from cyamelurate as a model for the oxygen-bearing functional groups of gC_3_N_4_ would suggest that these types of defects exhibit weak interactions with platinum. Later sections in the main text will discuss whether such interactions are sufficient for stabilizing metallic platinum catalysts.

# Characterization of compound **2**

For compound **2**, refined crystal data and atomic coordinates are tabulated in Table S8 and Table S9, respectively, while results from CHN elemental analyses as shown in Table S10 are largely consistent with those of the crystal structure solution. When the structure factor data were included for CheckCIF, alerts A and B were generated due to the residual electron density (Q-peaks) surrounding the two platinum positions. Such alerts are commonly reported^12^ especially in crystallographic data of (heavy) metal complexes having small or multiple crystalline domains.^13, 14^ However, in the case of compound **2**, a key rationale for these Q-peaks relates to the positional disorder of the two platinum centers. Some of such disorders (presumably static disorder) were modelled with multiple atomic positions having partial occupancies (as in the case with compound **1**). Specifically, disorder in the Pt2 position was modelled with Pt 2A, while Pt1A was introduced to model the positional disorder from thermal vibration of Pt1. The remaining residual densities adjacent to Pt1 and surrounding Pt2 are attributed to the heavy platinum atoms exhibiting unusually large thermal vibration arising from weak intermolecular interactions.^15^

Table S8 Crystal data and structural refinements for compound **2**.

| Empirical formula | C9 H18 N19 Pt1.46 |
| --- | --- |
| Formula weight | 1370.12 |
| Temperature [K] | 100 |
| Crystal system | Monoclinic |
| Space group (number) | P2_1_/c (14) |
| *a* [Å] | 3.8896(5) |
| *b* [Å] | 12.0637(15) |
| *c* [Å] | 48.095(6) |
| α [°] | 90 |
| β [°] | 91.605(3) |
| γ [°] | 90 |
| Volume [Å^3^] | 2255.9(5) Å^3^ |
| *Z* | 4 |
| *ρ*_calc_ [g cm^−3^] | 1.996 |
| *μ* [mm^−1^] | 9.121 |
| *F*(000) | 1277 |
| Crystal size [mm^3^] | 0.119×0.022×0.018 |
| Crystal colour | Colourless |
| Crystal shape | Needle |
| Radiation | Mo *K_α_* (λ=0.71073 Å) |
| 2θ range [°] | 1.889 to 28.34 |
| Index ranges | −5 ≤ h ≤ 5 −16 ≤ k ≤ 16 −64 ≤ l ≤ 64 |
| Reflections collected | 41302 |
| Independent reflections | 5598  *R*_int_ = 0.0563 *R*_sigma_ = 0.0369 |
| Completeness to θ = 25.242° | 99.6 |
| Data / Restraints / Parameters | 5598 / 2 / 282 |
| Goodness-of-fit on *F*^2^ | 1.391 |
| Final *R* indexes [*I*≥2σ(*I*)] | R_1_ = 0.0753, wR_2_ = 0.1591 |
| Final *R* indexes [all data] | R_1_ = 0.0776, wR_2_ = 0.1598 |
| Largest peak/hole [eÅ^−3^] | 5.262/–5.220 |

Table S9 Atomic coordinates, equivalent displacement parameters (Å^2^) and occupancies for compound **2**.

| Atom | Site | x | y | z | U_eq_ | Occ |
| --- | --- | --- | --- | --- | --- | --- |
| Pt1 | 4e | 0.66780(13) | 0.025069 | 0.67143(2) | 0.01270(16) | 0.947(6) |
| Pt1A | 4e | 0.665(4) | 0.076620 | 0.6706(3) | 0.01270(16) | 0.0321(16) |
| Pt2 | 2a | 0 | 0.500000 | 0.500000 | 0.0131(3) | 0.929(8) |
| Pt2A | 4e | -0.005(6) | 0.521(2) | 0.4856(8) | 0.0131(3) | 0.0206(19) |
| N1 | 4e | 0.634(3) | 0.3240(10) | 0.7437(2) | 0.023(2) | 1 |
| N2 | 4e | 0.773(3) | 0.6670(9) | 0.6302(2) | 0.017(2) | 1 |
| N3 | 4e | 0.500(3) | 0.3998(9) | 0.6760(2) | 0.017(2) | 1 |
| N4 | 4e | 0.786(3) | 0.5751(8) | 0.6722(2) | 0.014(2) | 1 |
| N5 | 4e | 0.496(3) | 0.4953(9) | 0.6334(2) | 0.016(2) | 1 |
| N6 | 4e | 0.231(3) | 0.3195(9) | 0.6377(2) | 0.015(2) | 1 |
| N7 | 4e | 0.747(3) | 0.7529(9) | 0.5864(2) | 0.020(2) | 1 |
| N8 | 4e | 0.486(3) | 0.5863(9) | 0.5902(2) | 0.019(2) | 1 |
| N9 | 4e | 0.217(3) | 0.4166(9) | 0.5938(2) | 0.018(2) | 1 |
| N10 | 4e | -0.040(3) | 0.2440(10) | 0.5997(2) | 0.023(3) | 1 |
| N11 | 4e | -0.039(3) | 0.5411(11) | 0.5410(2) | 0.029(3) | 1 |
| N12 | 4e | 0.199(3) | 0.3466(10) | 0.5102(3) | 0.044 | 1 |
| N13 | 4e | 0.850(3) | 0.1789(10) | 0.6832(2) | 0.044 | 1 |
| N14 | 4e | -0.259(4) | 0.2402(13) | 0.5508(3) | 0.044 | 1 |
| N15 | 4e | 0.550(3) | 0.0828(11) | 0.6325(2) | 0.028(3) | 1 |
| N16 | 4e | 0.497(3) | -0.1279(11) | 0.6605(2) | 0.043 | 1 |
| N17 | 4e | 0.779(3) | -0.0339(11) | 0.7107(2) | 0.043 | 1 |
| N18 | 4e | 1.061(3) | 0.9163(10) | 0.6061(2) | 0.043 | 1 |
| N19 | 4e | 0.781(3) | 0.4833(9) | 0.7136(2) | 0.022(2) | 1 |
| C1 | 4e | 0.693(3) | 0.5797(9) | 0.6455(2) | 0.034 | 1 |
| C2 | 4e | 0.912(4) | 0.8367(13) | 0.5981(3) | 0.034 | 1 |
| C3 | 4e | 0.674(3) | 0.6686(10) | 0.6029(2) | 0.034 | 1 |
| C4 | 4e | 0.685(3) | 0.4865(10) | 0.6867(2) | 0.036(3) | 1 |
| C5 | 4e | 0.689(3) | 0.3965(11) | 0.7283(3) | 0.025(3) | 1 |
| C6 | 4e | 0.407(3) | 0.4023(10) | 0.6491(2) | 0.037 | 1 |
| C7 | 4e | 0.398(3) | 0.4998(11) | 0.6051(2) | 0.037 | 1 |
| C8 | 4e | 0.142(4) | 0.3296(11) | 0.6097(3) | 0.037 | 1 |
| C9 | 4e | -0.144(4) | 0.2472(12) | 0.5732(3) | 0.026(3) | 1 |

Table S10 CHN elemental analyses of compound **2** as mass percentage and as molar ratio.

| Element | Mass percentage | Molar ratio | Molar ratio (normalized to 9 C) |
| --- | --- | --- | --- |
| C | 15.07 | 1.255 | 9 |
| N | 35.57 | 2.539 | 18 |
| H | 3.022 | 3.0215 | 22 |

Figure S9 FTIR spectrum of compound **2** compared with spectra of potassium melonate and the platinum precursor [(NH_3_)_4_Pt^II^](NO_3_)_2_.

Figure S10 XPS spectra of compound **2** in the C1s, N1s, O1s and Pt4f regions; fitting parameters are tabulated in Table S11.

Table S11 Peak fitting parameters of the spectra (Figure S10) of compound **2**.

| Element & peak number | BE (eV) | FWHM | Integral | χ^2^ |
| --- | --- | --- | --- | --- |
| C1s – peak 1 | 284.7 | 1.34 | 7338 | 1.14 |
| C1s – peak 2 | 286.2 | 1.56 | 3320 |  |
| C1s – peak 3 | 288.0 | 1.71 | 2984 |  |
| N1s – peak 1 | 398.3 | 1.48 | 6009 | 0.25 |
| N1s – peak 2 | 400.0 | 1.73 | 4477 |  |
| O1s – peak 1 | 532.0 | 1.66 | 4323 | 1.02 |
| O1s – peak 2 | 533.4 | 1.57 | 2572 |  |
| Pt4f – peak 1 | 73.2 | 1.49 | 14369 | 8.74 |

# Characterization of solid residues of potassium melonate and K_2_PtCl_4_ & Pt(NO_3_)_2_

As in the case of potassium cyamelurate, no precipitate was formed upon mixing aqueous solutions of potassium melonate with either K_2_PtCl_4_ or Pt(NO_3_)_2_, suggesting that a (poorly soluble) coordination polymer was not formed. However, since Pt–melonate coordinative bond might nevertheless be present, these two mixtures were evaporated to dryness and the solid residues were analyzed by XRD, FTIR and XPS, the results of which are shown in Figure S11 and Figure S12.

Figure S11 Characterization by a) XRD and b) FTIR of the solid residues obtained by mixing aqueous solution of potassium melonate and of K_2_PtCl_4_ or Pt(NO_3_)_2_, followed by evaporation to dryness.

While the residue from K_2_PtCl_4_ is amorphous, the one from Pt(NO_3_)_2_ yielded a diffraction peak at around 27.3° 2θ, which was also observed in the residue of potassium cyamelurate and Pt(NO_3_)_2_ (see Figure S5). Hence, this phase is attributed to some hydrolytic decomposition product of platinum nitrate (i.e. PtO or PtO_2_ that was mentioned above) rather than crystals of Pt–melonate complex. For further evidence indicative of Pt–melonate coordination, we examined the FTIR bands of the residues, focusing on whether there are wavenumber shifts when compared to the precursors. Key vibrational bands are tabulated in Table S12, which includes the C≡N stretching vibration from the cyanamide moiety at 2167 cm^-1^, heptazine vibrational modes at 1428 and 1634 cm^-1^, and the heptazine out-of-plane vibration at 792 cm^-1^.

Table S12 Wavenumbers in cm^-1^ for the IR bands corresponding to the C≡N stretching vibration, the heptazine vibration modes and the heptazine out-of-plane vibration for potassium melonate, compound **2** and the two residues from potassium melonate and K_2_PtCl_4_ and Pt(NO_3_)_2_.

| Entry | Sample | C≡N vibration | Heptazine vibrational modes | Heptazine out-of-plane vibration |
| --- | --- | --- | --- | --- |
| 1 | Potassium melonate | 2167 | 1634 / 1428 | 792 |
| 2 | Compound **2** | 2164 | 1625 / 1416 | 795 |
| 3 | Potassium melonate + K_2_PtCl_4_ | 2167 | 1616 / 1384 | 800 |
| 4 | Potassium melonate + Pt(NO_3_)_2_ | 2169 | 1635 / 1408 | 793 |

Based on published analyses of melonate complexes, coordination generally takes place between the metal and the cyanamide moiety, leading to wavenumber shift for the cyanamide’s C≡N vibration to values within the range of 2184–2157 cm^-1^.^16-20^ However, the values in Table S12 show only small shift of ≤2 cm^-1^ when comparing the two residues with potassium melonate, smaller than that of compound **2** where only second coordination sphere was observed, suggesting that cyanamide is not involved in any coordination bonding. Relatively more prominent shifts could be observed for the bands associated with the heptazine core, especially for the residue from K_2_PtCl_4_ as shown in entry 3 for all three vibrational bands. For the residue from Pt(NO_3_)_2_, only the band at 1408 cm^-1^ was significantly shifted compared to that of potassium melonate. If these shifts were to correspond to Pt–melonate complexation, these results would suggest that coordination occurred without involvement of the cyanamide, which is unlikely given that, as mentioned above, published crystal structures of metal melonate generally show coordination between –NCN and the metal centers. Moreover, other explanations could account for the shift of the vibrational bands related to the heptazine center. Firstly, any minor wavenumber shifts could be due to second-sphere coordination of the platinum complex, identical to the case of compound **2**. A second explanation is that these perceived shifts are the result of overlap with the bands of the platinum precursor, specifically overlap with the sharp bands of pure K_2_PtCl_2_ at 1592 and 1411 cm^‑1^, and the broad band around 1321 cm^-1^ of Pt(NO_3_)_2_. As such, neither XRD nor FTIR provide conclusive evidence of direct Pt–melonate coordination.

Turning to the XPS spectra and first discussing the results of the residue from melonate and K_2_PtCl_4_, the BE value of the Pt4f7/2 peak appears to shift upward by 0.3 eV while the Cl2p3/2 peak to lower value by 0.4 eV when compared to the pure K_2_PtCl_4_ (Table S1). Concurrently, when compared to potassium melonate^8^ and compound **2**, the BE of the C1s peak at 286.5 eV (attributed to the cyanamide moiety) has shifted to higher values by 0.3–0.4 eV while the N1s peak at 398.5 eV (assigned to the outer heptazine nitrogens and the cyanamide group) has BE increased by 0.2 eV. These shifts could be consistent with melonate donating electron density to the metal center as part of coordination and with platinum substituting its chloro ligand with heptazine molecules that are more electron withdrawing. Nevertheless, given that the magnitude of these shifts are equal to or smaller than the XPS’s spectral and energy resolution, one may argue that these results are inconclusive as to whether Pt–melonate complexation occurred, from which we surmise that any Pt–melonate coordination bond in this residue is likely to be very weak, if present at all.

The residue of melonate and Pt(NO_3_)_2_ exhibits similarly ambiguous results: the C1s peak at 286.3 eV and the N1s peak at 398.5 eV increased by ≤0.2 eV relative to the BE values of potassium melonate in reference ^8^ and compound **2**. In other words, since all shifts are of magnitudes within the spectrometer’s spectral/energy resolution, it is questionable whether these shifts are real or analysis uncertainties. On the other hand, for the Pt4f peaks at 73.0 and 74.9 eV, their BE values have decreased greater amounts of 0.3–0.5 eV relative to pure Pt(NO_3_)_2_ (Table S1), a result that indicates nitrate substitution with a ligand that is less electron-withdrawing, perhaps melonate. Nevertheless, as in the discussions regarding the XRD results, we cannot rule out the explanation that such shifts are attributed to (partial) hydrolysis of this platinum precursor to platinum oxides. Hence, these results are again inconclusive as to the formation of Pt–melonate complex, and that any Pt–melonate coordination bond is likely weak if present at all.

Figure S12 XPS spectra of the solid residue obtained by evaporating an aqueous solution of potassium melonate and either K_2_PtCl_4_ (top) or Pt(NO_3_)_2_ (bottom), with the fitting parameters tabulated in Table S13.

Table S13 Fitting parameters of the spectra in Figure S12. Spin-orbital coupling constant was constrained to 1.6 eV for fitting the Cl2p signals and to 2.8 eV for the K2p signals.

| Element & peak number | BE (eV) | FWHM | Integral | χ^2^ |
| --- | --- | --- | --- | --- |
| **K_2_PtCl_4_ + potassium melonate** | |  |  |  |
| C1s – peak 1 | 284.7 | 1.49 | 5070 | 0.69 |
| C1s – peak 2 | 286.5 | 1.66 | 4515 |  |
| C1s – peak 3 | 287.9 | 1.28 | 5236 |  |
| N1s – peak 1 | 398.5 | 1.56 | 18827 | 2.02 |
| N1s – peak 2 | 400.5 | 1.76 | 1919 |  |
| O1s – peak 1 | 531.1 | 2.23 | 2079 | 0.83 |
| O1s – peak 2 | 532.6 | 1.81 | 3665 |  |
| Pt4f7/2 – peak 1 | 72.9 | 1.47 | 15774 | 3.40 |
| K2p3/2 – peak 1 | 292.8 | 1.38 | 43033 | 4.58 |
| Cl2p3/2 – peak 1 | 198.2 | 1.19 | 17631 | 1.70 |
| **Pt(NO_3_)_2_ + potassium melonate** | |  |  |  |
| C1s – peak 1 | 284.7 | 1.51 | 5426 | 0.79 |
| C1s – peak 2 | 286.3 | 1.4 | 4634 |  |
| C1s – peak 3 | 288.0 | 1.44 | 5765 |  |
| N1s – peak 1 | 398.5 | 1.53 | 17154 | 1.21 |
| N1s – peak 2 | 400.5 | 1.9 | 2105 |  |
| N1s – peak 3 | 404.1 | 1.45 | 1621 |  |
| N1s – peak 4 | 406.8 | 1.38 | 1159 |  |
| O1s – peak 1 | 530.9 | 1.77 | 8265 | 0.87 |
| O1s – peak 2 | 532.4 | 1.75 | 20164 |  |
| Pt4f7/2 – peak 1 | 73.0 | 1.56 | 18743 | 6.27 |
| Pt4f7/2 – peak 2 | 74.9 | 1.32 | 25950 |  |
| K2p3/2 – peak 1 | 292.8 | 1.41 | 22255 | 1.85 |

# UV-Vis titration of K_2_PtCl_4_ and Pt(NO_3_)_2_ with potassium melonate

As in the case with the cyamelurate, UV-Vis titration was performed to determine whether melonate displaced the chloro and nitrato ligand of K_2_PtCl_4_ and Pt(NO_3_)_2_ within the aqueous solution phase, which would manifest as shifts in the absorption bands. UV-Vis spectra of these titration experiments are shown in Figure S13.

Figure S13 UV-Vis titration of potassium melonate solutions with K_2_PtCl_4_ (a-b) and with Pt(NO_3_)_2_ (c-d).

For the titration of K_2_PtCl_4_ (1 mL, 5.1 mM) in Figure S13a, mixtures 1–8 in the legend refer to addition of potassium melonate (4.5 mM) in 188 μL aliquots up to a total of 1504 μL for mixture 8 (i.e. 5.1 μmol of Pt^2+^ and a total of 6.8 μmol of melonate). Here, with increasing addition of potassium melonate, the bands of K_2_PtCl_4_ at 388 and 468 nm have lower intensities due to dilution, although some shifts seem to occur which could signify Pt–melonate complexation or may be due to a dilution effect. To clarify this, the absorption spectrum of potassium melonate was also examined by employing highly diluted solutions, owing to the large extinction coefficient of its UV-Vis band at 282 nm. These results are presented in Figure S13b, which shows the titration of potassium melonate (1 mL, 18 μM) whereby the plots labelled “mixtures 1–8” in the legend refer to addition of K_2_PtCl_4_ (20 μM) in 330 μL aliquots up to a total of 2640 μL for mixture 8 (i.e. 18 nmol of melonate and a total of 53 nmol of Pt^2+^). Here, no shifts in the melonate band is apparent, making it ambiguous as to whether Pt–melonate complexation was formed through the displacement of the chloro ligand by the melonate.

For the titration of Pt(NO_3_)_2_ (1 mL, 6.96 mM) in Figure S13c, mixtures 1–8 in the legend refer to addition of potassium melonate (18 mM) in 65 μL aliquots up to a total of 520 μL for mixture 8 (i.e. 6.96 μmol of Pt^2+^ and a total of 9.36 μmol of melonate). Here, increasing the amount of melonate led to emergence of its band at 282 nm that exhibited no wavelength shift, contrary to the expectation of peak shift should Pt–melonate complexation occur. For completion, we have also swapped the analyte and the titrant in Figure S13d, which shows the spectra for the titration of potassium melonate (1 mL,18 μM) with Pt(NO_3_)_2_. Here, the plots labelled as “mixtures 1–8” in the legend refer to addition of Pt(NO_3_)_2_ (174 μM) in 39 μL aliquots up to a total of 312 μL for mixture 8 (18 nmol of melonate and a total of 54 nmol of Pt^2+^). The melonate absorption band blue-shifted by 7 nm at the highest Pt(NO_3_)_2_ concentration in mixture 8, which could signify Pt–melonate complexation. However, this apparent shift might be due to superimposition with the broad absorption of Pt(NO_3_)_2_, the spectrum of which is plotted in orange in Figure S13d and spans across the ultraviolet range. Considering the alternative explanations possible, these titration results are inconclusive as to whether melonate displaced the chloro and nitrato ligands of K_2_PtCl_4_ and Pt(NO_3_)_2_ to yield Pt–melonate complexes in the solution phase.

# Product characterization from attempted complexation with melem in aqueous suspension

To determine whether Pt–melem complexation took place in the aqueous suspension of melem and the three soluble platinum precursors (method 1), the solid phases were characterized by XRD and FTIR, and the results are presented in Figure S14a-b.

Figure S14 a) XRD patterns and b) FTIR spectra of the solids recovered from aqueous solutions of [(NH_3_)_4_Pt^II^](NO_3_)_2_, K_2_PtCl_4_ and Pt(NO_3_)_2_ with melem suspended; also shown are the diffraction patterns and FTIR spectra of the precursors. c) Relative change of the Pt concentration in the supernatant before and after the attempted complexation.

From the XRD patterns of the cases when the platinum precursor is [(NH_3_)_4_Pt](NO_3_)_2_ or Pt(NO_3_)_2_, the main reflections match those melem hydrate as indicated by the hairlines drawn, indicating that this is the major phase. However, for the patterns of K_2_PtCl_4_ and Pt(NO_3_)_2_, a reflection is present at 28.1° 2θ, although this is not observed in melem (anhydrous and hydrate). As this reflection might originate from a Pt–melem complex, we attempted to confirm the presence of Pt–melem coordination bonds by examining their FTIR spectra, specifically for wavenumber shifts as compared with melem hydrate. As observed in Table S14 tabulating the wavenumbers of the prominent vibration modes of heptazine, the solid recovered from melem suspended in a solution of K_2_PtCl_4_ exhibited the largest wavenumber shifts for the three heptazine vibration modes, which could indicate Pt–melem complexation. Nevertheless, these results are insufficient as evidence to demonstrate direct Pt–melem bonding, considering that these shifts of ≤10 cm^-1^ may be related to other intermolecular interactions in melem hydrate, for example, second-sphere type PtCl_4_–melem coordination. Hence, for additional evidence for or against the presence of first-sphere Pt–melem coordination, we performed elemental quantification of the platinum solution before and after melem addition, in the expectation that the Pt concentration should decrease after contacting the suspended melem due to complexation and precipitation. These results are plotted in Figure S14c.

Table S14 Wavenumbers in cm^-1^ for the IR bands corresponding to the prominent vibrational modes of melem hydrate and for the solids recovered from aqueous solutions of [(NH_3_)_4_Pt^II^](NO_3_)_2_, K_2_PtCl_4_ and Pt(NO_3_)_2_ with melem suspended.

| Sample | Heptazine vibrational mode 1 | | Heptazine vibrational mode 2 | Heptazine out-of-plane vibration |
| --- | --- | --- | --- | --- |
| Melem hydrate | | 1601 | 1444 | 790 |
| Melem + [(NH_3_)_4_Pt](NO_3_)_2_ | | 1595 | 1440 | 790 |
| Melem + K_2_PtCl_4_ | | 1591 | 1435 | 796 |
| Melem + Pt(NO_3_)_2_ | | 1595 | 1435 | 790 |

In this figure panel, all Pt concentrations have been normalized to that before adding melem to form the suspension, that is, 100% represent the concentrations of the stock Pt solutions. As seen here, the concentration decreased by no more than 10% suggesting that the majority of the platinum remained in the solution while any Pt–melem complex formed (if at all) to be a minor phase. Note that an alternative interpretation of these results is that the decrease in Pt concentration in the supernatant can be attributed to physisorption of [(NH_3_)_4_Pt]^2+^ or [PtCl_4_]^2–^ complexes onto the hydrogen-bonded network of melem hydrate, rather than a first-sphere coordination. Thus, for addition evidence supporting each of these possibilities, the three solids were analyzed by XPS and their spectra are presented in Figure S15, while the fitting parameters tabulated in Table S15.

Figure S15 XPS spectra of the solids recovered from aqueous solutions of [(NH_3_)_4_Pt^II^](NO_3_)_2_, K_2_PtCl_4_ and Pt(NO_3_)_2_ with melem suspended; fitting parameters are tabulated in Table S15.

Table S15 Peak fitting parameters of the spectra in Figure S15

| Element & peak number | BE (eV) | FWHM | Integral | χ^2^ |
| --- | --- | --- | --- | --- |
| **[(NH_3_)_4_Pt](NO_3_)_2_ + melem** | |  |  |  |
| C1s – peak 1 | 284.6 | 1.69 | 4097 | 5.91 |
| C1s – peak 2 | 287.5 | 1.49 | 17213 |  |
| N1s – peak 1 | 398.0 | 1.41 | 23486 | 1.59 |
| N1s – peak 2 | 398.8 | 1.55 | 11752 |  |
| N1s – peak 3 | 400.4 | 1.36 | 3824 |  |
| O1s – peak 1 | 532.2 | 2.58 | 4134 | 0.68 |
| **K_2_PtCl_4_ + melem** | |  |  |  |
| C1s – peak 1 | 284.7 | 1.44 | 25585 | 3.87 |
| C1s – peak 2 | 286.2 | 1.72 | 8230 |  |
| C1s – peak 3 | 288.3 | 1.51 | 6167 |  |
| N1s – peak 1 | 398.3 | 1.41 | 4746 | 0.66 |
| N1s – peak 2 | 399.2 | 1.42 | 2376 |  |
| N1s – peak 3 | 400.7 | 1.47 | 870 |  |
| O1s – peak 1 | 531.8 | 1.38 | 9855 | 1.57 |
| O1s – peak 2 | 533.2 | 1.59 | 12098 |  |
| Pt4f7/2 – peak 1 | 72.8 | 1.98 | 3464 | 3.36 |
| Cl2p3/2 – peak 1 | 198.2 | 1.93 | 622 | 2.07 |
| **Pt(NO_3_)_2_ + melem** | |  |  |  |
| C1s – peak 1 | 284.4 | 1.58 | 5203 | 4.08 |
| C1s – peak 2 | 285.4 | 1.58 | 1355 |  |
| C1s – peak 3 | 287.8 | 1.49 | 18341 |  |
| N1s – peak 1 | 398.2 | 1.43 | 24411 | 1.37 |
| N1s – peak 2 | 399.1 | 1.57 | 12214 |  |
| N1s – peak 3 | 400.6 | 1.38 | 4063 |  |
| O1s – peak 1 | 531.9 | 2.33 | 10841 | 1.37 |
| Pt4f7/2 – peak 1 | 73.0 | 1.87 | 2885 | 2.07 |
| Pt4f7/2 – peak 2 | 74.8 | 1.66 | 5123 |  |

A notable result from the XPS is the absence of Pt4f signal for the melem suspended in the [(NH_3_)_4_Pt^II^](NO_3_)_2_ solution, that is, the insoluble melem contained platinum of an amount below the detection limit of XPS. This result appears contradictory to the findings of Figure S14c, where the decrease in Pt concentration after melem addition was interpreted as either melem-platinum coordination or at least the adsorption of the tetraammineplatinum complex. However, considering that the supernatant Pt concentration to decrease by only ≈10%, one explanation is that the interaction between [(NH_3_)_4_Pt^II^]^2–^ and melem is quite weak, causing the amount of Pt adsorbed from solution onto melem to be so small so as to not only introduce significant error in the elemental quantification but also indetectable by XPS.

As for the melem in aqueous K_2_PtCl_4_, any differences in BE values of the Pt4f and Cl2p3/2 peaks from those of pure K_2_PtCl_4_ (Table S1) are within the spectral resolution of XPS spectrometer, while the BE of the N1s and O1s peaks are identical to those of pure melem hydrate from our previous publication.^8^ Notably, the presence of the Cl2p signal suggests platinum to remain as [PtCl_4_]^2-^ that is physisorbed onto melem hydrate, causing the FTIR band shifts mentioned above. Lastly, in the case of melem in Pt(NO_3_)_2_, the results are similar: all N1s and O1s signals have identical BE to those of pure melem, while the first Pt4f7/2 peak differs from pure Pt(NO_3_)_2_ by 0.3 eV. Given that all BE changes are within the resolution of XPS spectrometer and are thus not statistically significant, results from XPS do not support the presence of first-sphere Pt–melem coordination. Combining all of the above results, any interaction between melem and platinum, either directly or via second-sphere coordination (i.e. between melem and either [(NH_3_)_4_Pt]^2+^ or [PtCl_4_]^2–^), appears weak (if present at all) so as to be nearly undetectable by the analytical techniques above.

# Product characterization from attempted complexation with melem in DMSO solution

The following describes the experimental details for the UV-Vis titration of melem in DMSO. [(NH_3_)_4_Pt^II^](NO_3_)_2_ was dissolved in DMSO at a concentration of 2.03 mM, and a specific volume was placed into a quartz cuvette to collect its UV-Vis spectrum, which is shown as the gray plot in Figure S16a. Separately, a solution of melem in DMSO was prepared at a concentration of 5.97 mM, and an aliquot of this melem solution was taken such that the molar amount of melem is equal to that of the platinum salt in the cuvette. This aliquot was diluted with DMSO to 1 mL and added to the cuvette to record the UV-Vis spectrum. This process was repeated such that the spectra in Figure S16a are of DMSO with molar ratio of melem : [(NH_3_)_4_Pt^II^](NO_3_)_2_ being 1:1, 2:1, 3:1 … up to 8:1. As observed in the inset of this figure, with increasing addition of the melem-DMSO solution to the tetraamineplatinum-DMSO solution, two UV bands with increasing intensity emerged at 302 and 315 nm. Since these two bands have undiscernible wavelength shift relative to the pure melem solution (plotted in orange), we infer that Pt–melem interaction is insignificant within DMSO.

A similar titration was performed For K_2_PtCl_4_ as follows. A solution of this platinum precursor in DMSO of concentration 4.76 mM was first prepared, and a specific volume was placed into a quartz cuvette to record its UV-Vis spectrum (red plot in Figure S16b). Separately, a solution of melem in DMSO of concentration 3.55 mM was prepared and an aliquot of this melem solution was taken such that the molar amount of melem is equivalent to the molar amount of K_2_PtCl_4_ in the cuvette. This aliquot was diluted with DMSO to 1 mL and added to the cuvette containing the K_2_PtCl_4_ solution, and the UV-Vis spectrum was recorded again. This addition process was repeated such that the spectra in Figure S16b are of DMSO with molar ratio of melem : K_2_PtCl_4_ being 1:1, 2:1, 3:1 and 4:1. Here, the main UV-Vis band at 334 nm originates from K_2_PtCl_4_ in the DMSO solution, and its intensity decreases with increasing addition of melem-DMSO solution due to dilution. However, since a change in the wavelength of this band is not discernible, we also inferred that melem did not displace the chloro ligand of [PtCl_4_]^2-^ in the DMSO solution, that is, any Pt–melem complexation occurring is at an indetectable amount, if at all.

Figure S16 UV-Vis spectra for titration in DMSO of melem and the platinum precursor, a) [(NH_3_)_4_Pt^II^](NO_3_)_2_ and b) K_2_PtCl_4_. Insets of both panels show the magnification of the main absorption band(s).

While the analyses of the DMSO solutions above did not suggest the presence of Pt–melem complex, for completion, we also analyzed any precipitation that formed for possible occurrence of Pt–melem coordination. In these DMSO solutions containing melem and either [(NH_3_)_4_Pt](NO_3_)_2_ or K_2_PtCl_4_, exposure to the atmosphere eventually leads to small amount of white precipitates. However, as shown in Figure S17a, their XRD patterns match that of melem hydrate, indicating this to be the major phase. In contrast, no crystalline phase corresponding to Pt-melem complex is detectable. Hence, these precipitates were analyzed by FTIR spectroscopy, and the results are shown in Figure S17b.

Figure S17 Characterization of the precipitates from DMSO solutions of melem and either [(NH_3_)_4_Pt](NO_3_)_2_ or K_2_PtCl_4_ by: a) XRD, and b) FTIR. Also shown in a) are the experimental pattern of melem hydrate (labelled with “exp”) as well as the pattern calculated from the crystallographic information file^21^ (labelled with “CIF”). Likewise, panel b) also shows the spectra of all reactant/reagent as reference, including the spectrum of DMSO that was obtained from SDBS (Spectral Database for Organic Compounds) and digitalized using OriginPro 2024b.

Based on the publication on Ag-melem complex,^22^ the wavenumbers of the vibration modes of heptazine shift by 20–30 cm^-1^ upon silver complexation when compared to non-coordinated melem. However, for the two precipitates here, no shift in the relevant vibrational bands is discernible from their FTIR spectra. Any change in wavenumber of these bands, as tabulated in Table S16, was found to be minor, and thus inconclusive as to whether Pt–melem coordination is present.

Table S16 Wavenumbers in cm^-1^ for the IR bands corresponding to the prominent vibrational modes of melem hydrate and for the precipitates from DMSO solutions of melem and either [(NH_3_)_4_Pt^II^](NO_3_)_2_ or K_2_PtCl_4_.

| Sample | Heptazine vibrational mode 1 | | Heptazine vibrational mode 2 | Heptazine out-of-plane vibration |
| --- | --- | --- | --- | --- |
| Melem hydrate | | 1601 | 1444 | 790 |
| Melem + [(NH_3_)_4_Pt](NO_3_)_2_ | | 1603 | 1459 | 793 |
| Melem + K_2_PtCl_4_ | | 1591 | 1434 | 796 |

For completion, we performed XPS analysis on the precipitate obtained from the DMSO solution of melem and K_2_PtCl_4_, focusing on this platinum precursor due to the greater likelihood of melem displacing the chloro ligand, as explained in the main text. The spectra are shown in Figure S18 with the fitting parameters tabulated in Table S17. Neither potassium nor chlorine was detected, while the fitting results in the C1s, N1s and O1s regions show that all signals have BE values identical (within the spectrometer’s spectral resolution) to our previous analysis of melem hydrate.^8^ A minute amount of platinum was detected, and its BE value of 73.0 eV is in the range expected of Pt^2+^ and is around 0.4 eV larger than that of pure K_2_PtCl_4_ (Table S1), all of which could indicate that some Pt centers have their chloro ligand substituted for melem. Nevertheless, one cannot rule out alternative explanations for these BE shifts, for example, that the Pt–melem interactions are in the second coordination sphere. Combining all of the results above, the evidences are ambiguous as to whether solubilization in DMSO led to Pt–melem complexation, regardless of whether the Pt precursor is [(NH_3_)_4_Pt^II^](NO_3_)_2_ or K_2_PtCl_4_, leading to the conclusion that the interactions between melem and platinum (if present at all) may be too weak to be definitively detected.

Figure S18 XPS spectra of the precipitate obtained from DMSO solution of melem and K_2_PtCl_4_ in the C1s, N1s, O1s, Pt4f and Cl2p regions; fitting parameters are Table S17.

Table S17 Peak fitting parameters of the spectra (Figure S18) of the precipitate obtained from DMSO solution of melem and K_2_PtCl_4_.

| Element & peak number | BE (eV) | FWHM | Integral | χ^2^ |
| --- | --- | --- | --- | --- |
| C1s – peak 1 | 284.7 | 1.44 | 18137 | 2.79 |
| C1s – peak 2 | 286.1 | 1.34 | 4996 |  |
| C1s – peak 3 | 288.1 | 1.89 | 9099 |  |
| N1s – peak 1 | 398.3 | 1.38 | 9238 | 1.02 |
| N1s – peak 2 | 399.2 | 1.57 | 4620 |  |
| N1s – peak 3 | 400.7 | 1.45 | 1550 |  |
| O1s – peak 1 | 531.9 | 1.46 | 6949 | 0.93 |
| O1s – peak 2 | 533.3 | 1.66 | 9581 |  |
| Pt4f – peak 1 | 73.0 | 2.00 | 510 | 0.98 |

# Characterization of Pt–gC_3_N_4_

Pristine melon-M, melon-U and PHI were characterized by XRD and FTIR, and the results shown in Figure S19 (black plots) are consistent with those in the literature.^23, 24^ No change was observed after 0.2 wt% of platinum loading (red plots), indicating that the support did not undergo structural changes. Negligible change was also observed following their use as transfer hydrogenation catalysts (blue plots), from which we conclude that these support materials are stable in this reaction.

Figure S19 a-c) XRD patterns and d-f) FTIR spectra of the three gC_3_N_4_ samples without and with platinum loaded at 0.2 wt%. Also shown are the XRD patterns and FTIR spectra of the samples recovered after their use as transfer hydrogenation catalyst.

Note that the XRD reflections of platinum are absent due to the low loading of 0.2 wt%, although elemental analysis (see main text) confirmed the presence of platinum species on the support. These platinum species could also be visualized by scanning transmission electron microscope in high-angle annular dark-field mode (HAADF-STEM), which we employed for the selected samples of Pt–Melon-M and Pt–PHI. The microscope images in Figure S20 show the particles of the heavy Pt species as bright spots, which have a diameter in the range of 1–5 nm.

Figure S20 HAADF-STEM images of a) Pt–Melon-M and b) Pt–PHI, with the bright spots corresponding to the heavy platinum species annotated in terms of their longest dimension.

Chemical analyses of the three platinized gC_3_N_4_ samples were undertaken using XPS, and the spectra in the C1s and N1s regions (Figure S21) are consistent with those in the literature.^8^ However, signal in the Pt4f region cannot be discerned from the noise due to the low loading of 0.2 wt%, (analogous to the absence of Pt reflections in their XRD patterns) and therefore could not be peak-fitted for speciation.

Figure S21 XPS spectra in the C1s, N1s and Pt4f regions for the three gC_3_N_4_ samples platinized at 0.2 wt% loading. Fitting parameters are tabulated in Table S18.

Table S18 Fitting parameters for the XPS spectra of Pt–gC_3_N_4_ (0.2 wt%) in Figure S21.

| Element & peak number | BE (eV) | FWHM | Integral | χ^2^ |
| --- | --- | --- | --- | --- |
| **Pt–melon-M (0.2 wt%)** |  |  |  |  |
| C1s – peak 1 | 284.8 | 1.40 | 19381 | 2.71 |
| C1s – peak 2 | 285.9 | 1.30 | 2851 |  |
| C1s – peak 3 | 288.2 | 2.43 | 7699 |  |
| N1s – peak 1 | 398.3 | 1.28 | 5351 | 0.18 |
| N1s – peak 2 | 399.5 | 1.50 | 1377 |  |
| N1s – peak 3 | 400.8 | 1.32 | 777 |  |
| **Pt–melon-U (0.2 wt%)** |  |  |  |  |
| C1s – peak 1 | 284.7 | 1.64 | 6856 | 0.64 |
| C1s – peak 2 | 286.1 | 1.44 | 1629 |  |
| C1s – peak 3 | 288.0 | 1.98 | 3163 |  |
| N1s – peak 1 | 398.3 | 1.42 | 2468 | 0.14 |
| N1s – peak 2 | 399.6 | 1.81 | 705 |  |
| N1s – peak 3 | 401.0 | 1.03 | 248 |  |
| **Pt–PHI (0.2 wt%)** |  |  |  |  |
| C1s – peak 1 | 284.8 | 1.32 | 23133 | 3.87 |
| C1s – peak 2 | 286.2 | 2.15 | 7973 |  |
| C1s – peak 3 | 288.5 | 1.36 | 5207 |  |
| N1s – peak 1 | 398.1 | 1.32 | 4000 | 0.12 |
| N1s – peak 2 | 398.9 | 1.36 | 967 |  |
| N1s – peak 3 | 400.7 | 1.36 | 695 |  |
| K2p – peak 1* | 292.6 | 1.25 | 2320 | 1.51 |

*Spectrum (Figure S22) fitted with a spin-orbital coupling energy difference of 2.8 eV.

Figure S22 XPS spectrum of Pt-PHI in the K2p region.

To ascertain the oxidation state of the Pt species, another sample of melon-M was platinized at a higher loading of 3.0 wt%, and the XPS spectra (Figure S23) clearly show the presence of metallic Pt^0^ based on the Pt4f_7/2_ and Pt4f_5/2_ signals at BE 70.7 eV and 74.1 eV, respectively. However, the Pt4f_7/2_ and Pt4f_5/2_ signals at BE 72.4 and 75.9 eV can also be observed and are consistent with Pt^2+^ species that are thought to arise from charge transfer to the support^25^ or from the platinum species anchoring to defects, such as oxygen-based functional groups^26^ or nitrogen vacancy.^27^ Presently, it is unclear whether the presence of such high valent platinum is evidence for metal-support interactions although, as is evident from this work, these interactions are insufficiently strong to immobilize platinum particles.

Figure S23 XPS of melon-M platinized at a higher loading of 3 wt%; fitting parameters are tabulated in Table S19.

Table S19 Fitting parameters for the XPS spectra of Pt–melon-M (3 wt%) in Figure S23.

| Element & peak number | BE (eV) | FWHM | Integral | χ^2^ |
| --- | --- | --- | --- | --- |
| C1s – peak 1 | 284.8 | 1.40 | 14629 | 1.36 |
| C1s – peak 2 | 286.1 | 1.21 | 2506 |  |
| C1s – peak 3 | 288.1 | 1.52 | 8165 |  |
| N1s – peak 1 | 398.4 | 1.30 | 8972 | 0.38 |
| N1s – peak 2 | 399.7 | 1.84 | 2852 |  |
| N1s – peak 3 | 401.1 | 1.04 | 640 |  |
| Pt4f7/2 – peak 1 | 70.7 | 1.50 | 1597 | 0.96 |
| Pt4f7/2 – peak 2 | 72.4 | 2.20 | 1246 |  |

Pt-melon-M samples after transfer hydrogenation catalysis were recovered by centrifugation, washed with water, and then dried for characterization by XPS. The spectra are shown in Figure S24 and the fitting results tabulated in Table S20. The binding energies of all synthetic peaks have negligible change compared to the values of the samples before transfer hydrogenation, indicative of catalyst stability for this reaction at least in terms of negligible chemical change. Nevertheless, for Pt–melon-M with 3 wt% loading after transfer hydrogenation, the two platinum peaks corresponding to Pt^0^ and Pt^2+^ (at BE 71.0 and 72.4 eV respectively) has a peak integral ratio of 1.6 (Pt^0^:Pt^2+^ of 197:124 from Table S20). This represents an increase compared to the ratio of 1.3 (Pt^0^:Pt^2+^ of 1597:1246 from Table S19) of the pristine catalyst, which suggests greater quantity of Pt^0^ likely due to Pt^2+^ reduction over the course of the transfer hydrogenation. The poorer signal-to-noise in the Pt4f region for the spent Pt-Melon-M (3 wt%) as compared to that of the pristine sample in Figure S23 suggests partial leaching of this metal from the support, as consistent with elemental analyses by AES.

Figure S24 XPS spectra of Pt-Melon-M after transfer hydrogenation catalysis; fitting parameters are tabulated in Table S20.

Table S20 Fitting parameters for the XPS spectra in Figure S24 of Pt–melon-M after transfer hydrogenation catalysis.

| Element & peak number | BE (eV) | FWHM | Integral | χ^2^ |
| --- | --- | --- | --- | --- |
| **Pt–melon-M (0.2 wt%) after transfer hydrogenation** | | |  |  |
| C1s – peak 1 | 284.7 | 1.90 | 1181 | 0.50 |
| C1s – peak 2 | 285.7 | 1.93 | 359 |  |
| C1s – peak 3 | 288.0 | 2.10 | 1048 |  |
| N1s – peak 1 | 398.2 | 1.62 | 988 | 0.19 |
| N1s – peak 2 | 399.4 | 1.42 | 450 |  |
| N1s – peak 3 | 400.7 | 1.43 | 198 |  |
| **Pt–melon-M (3 wt%) after transfer hydrogenation** | | |  |  |
| C1s – peak 1 | 284.8 | 1.64 | 1619 | 1.08 |
| C1s – peak 2 | 286.0 | 1.38 | 734 |  |
| C1s – peak 3 | 288.3 | 1.94 | 2644 |  |
| N1s – peak 1 | 398.5 | 1.58 | 2629 | 0.47 |
| N1s – peak 2 | 399.5 | 1.72 | 1875 |  |
| N1s – peak 3 | 400.8 | 1.75 | 866 |  |
| Pt4f7/2 – peak 1 | 71.0 | 1.79 | 197 | 0.26 |
| Pt4f7/2 – peak 2 | 72.8 | 2.48 | 124 |  |

# Characterization of Pt–TiO_2_ catalysts

Commercially available TiO_2_ nanoparticles were used as support for preparing Pt–TiO_2_ at 0.2 wt% platinum loading for comparison with gC_3_N_4_, and the characterization results by XRD and FTIR are shown in Figure S25a-b.

Figure S25 Characterization by XRD (a, c) and FTIR (b, d) of Pt–TiO_2_ at platinum loading of 0.2 wt% (a, b) and 3.0 wt% (c, d).

Reflections at 27.4°, 36.2 ° and 41.3° 2θ are characteristics of the rutile phase, though reflections allowing identification of the platinum species are not discernible at 0.2 wt% loading, as in the case above for the gC_3_N_4_. In the FTIR spectra, the band at 670 cm^-1^ has been assigned to Ti–O–Ti vibration while the bands at 3300 and 1640 cm^-1^ to the vibration modes of adsorbed water. After catalytic transfer hydrogenation, only minor changes in the XRD pattern and FTIR spectrum can be observed, indicating stability of the support. In the FTIR, some peaks in the range 2800–3000 cm^-1^ could be attributed to the strong adsorption of substrate or product on the TiO_2_ surface. To directly ascertain the presence of platinum species on TiO_2_, they were visualized with HAADF-STEM, as shown in the microscopy images of Figure S26. Here, the platinum species are observable as bright spots with diameter in the range of 1–6 nm against the lighter background of TiO_2_.

Figure S26 HAADF-STEM images of Pt–TiO_2_ at a) low and b) high magnification, with the bright spots corresponding to the heavy platinum species annotated in terms of their longest dimension.

XPS spectra of TiO_2_ and Pt–TiO_2_ (0.2 wt% and 3 wt% Pt) are shown in Figure S27, while fitting parameters are provided in Table S21. Note that the Ti2p_1/2_ and Ti2p_3/2_ peaks were fitted individually without constraint, since the former is broadened by the Coster-Kronig effect^28^ and thus does not conform to the expected integral of 50% of that of the Ti2p_3/2_ peak. Note also that the Pt4f7/2 signals, although they are discernible as highlighted in orange in the figure, are not fitted due to their poor signal-to-noise.

The BE of the Ti2p3/2 signal at 458.5 eV and the O1s signal at 529.6 eV are consistent with those previously reported for TiO_2_,^29^ and are similar for the three samples, indicating that no chemical change to the support occurred following platinization. The second broad signal in the O1s region can be assigned to physisorbed water or surficial hydroxyl.^6^ In the Ti2p region of pure TiO_2_, an addition peak at 460.6 eV might be attributed to anionic oxide overlayer^30^ that reduces electron density of the Ti^4+^ underneath, yielding a signal with a larger BE. However, this additional signal is negligible for the two Pt–TiO_2_, suggesting that the platinization somehow resulted in uniform electron density or oxidation state of the underlying titanium centers. In the Pt4f region, the dominant signal is at around 73.8 eV, which is unlikely to be a Pt4f7/2 signal from high-valence platinum, since the corresponding Pt4f5/2 signal (which would have 3.4 eV higher BE ) is absent. Thus, we attribute this to be the Pt4f5/2 signal accompanying the discernible Pt4f7/2 signal(s) in the BE range 68.9–71.6 eV (highlighted in orange), which would correspond to Pt^0^, although it is presently unclear why these two signals do not conform to the expected integral ratio of 4:3. After transfer hydrogenation catalysis by Pt–TiO_2_ (3 wt%), all of its XPS signals appear largely unchanged signifying the chemical stability of this support. One difference is the appearance of an O1s signal at 533.2 eV that has been attributed to (strongly physisorbed) water,^6^ which in the present case originated from the catalytic reaction and incomplete removal during drying.

Figure S27 XPS spectra in the Ti2p, O1s and Pt4f regions of TiO_2_ and Pt–TiO_2_ (0.2 wt% and 3 wt% Pt). Fitting parameters are tabulated in Table S21.

Table S21 Peak fitting parameters of the spectra (Figure S27) of pristine TiO_2_ and TiO_2_ with platinum loaded at 0.2 and 3 wt%. Note that the Ti2p_1/2_ and Ti2p_3/2_ peaks were fitted separately (see accompanying text).

| Element & peak number | BE (eV) | FWHM | Integral | χ^2^ |
| --- | --- | --- | --- | --- |
| **TiO_2_** |  |  |  |  |
| Ti2p – peak 1 | 458.5 | 1.28 | 7675 | 0.98 |
| Ti2p – peak 2 | 460.6 | 2.20 | 1248 |  |
| Ti2p – peak 3 | 464.2 | 1.95 | 3838 |  |
| O1s – peak 1 | 529.7 | 1.32 | 8506 | 2.00 |
| O1s – peak 2 | 531.7 | 2.51 | 11583 |  |
| **Pt–TiO_2_ (0.2 wt%)** |  |  |  |  |
| Ti2p – peak 1 | 458.4 | 1.33 | 9367 | 4.29 |
| Ti2p – peak 2 | 464.1 | 2.05 | 4855 |  |
| O1s – peak 1 | 529.6 | 1.26 | 8666 | 3.62 |
| O1s – peak 2 | 531.9 | 2.81 | 20980 |  |
| **Pt–TiO_2_ (3 wt%)** |  |  |  |  |
| Ti2p – peak 1 | 458.6 | 1.37 | 7274 | 2.07 |
| Ti2p – peak 2 | 464.3 | 2.11 | 3637 |  |
| O1s – peak 1 | 529.7 | 1.36 | 6256 | 4.81 |
| O1s – peak 2 | 532.2 | 2.75 | 19437 |  |
| **Pt–melon-M (3 wt%) after transfer hydrogenation** | | |  |  |
| Ti2p – peak 1 | 458.4 | 1.58 | 3573 | 1.46 |
| Ti2p – peak 2 | 464.0 | 2.21 | 2064 |  |
| O1s – peak 1 | 529.6 | 1.71 | 4494 | 0.57 |
| O1s – peak 2 | 531.7 | 1.66 | 1969 |  |
| O1s – peak 3 | 533.2 | 1.84 | 1205 |  |

# Transfer hydrogenation of nitrobenzene into aniline as catalyzed by Pt–gC_3_N_4_ & Pt–TiO_2_

Transfer hydrogenation of nitrobenzene using formic acid as hydrogen source was used to assess the stability of the three Pt–gC_3_N_4_ catalysts, and are compared with Pt–TiO_2_. This reaction is depicted in Figure S28a)i). While our focus is on platinum leaching from the support, for comprehensiveness, reaction outcomes were assessed using ^1^H NMR for product identification (Figure S28b-c) and gas chromatography (GC) for quantification.

Figure S28 a) i) Schematic of transfer hydrogenation of nitrobenzene to aniline using formic acid as hydrogen source; ii) schematic of formanilide formation from aniline and formic acid. b) ^1^H NMR spectra in CDCl_3_ of the reaction mixture before (T = 0 h) and after the transfer hydrogenation (T= 72 h) for the case with and without platinized gC_3_N_4_. For reference, the spectra of precursors (formic acid and nitrobenzene) and the hydrogenation product (aniline) are also plotted. c) ^1^H NMR spectra in D_2_O of the reaction mixture containing only aniline and formic acid (no catalyst) before and after heating, respectively labelled as T = 0 h and T = 72 h. Inset shows the structure of formanilide and the shaded regions of the molecule and the spectrum are color-coded to indicate signal assignments.

We first discuss the reaction outcome based on the ^1^H NMR spectra of Figure S28b, which employed CDCl_3_ to solubilize both the precursors and the product(s), but form a two-phase mixture with the reaction solvent, water. As such, these spectra are not quantitative due to the precursors and product(s) having different relative miscibility in water and chloroform. The black and red plots show the spectra of the reaction without any catalyst initially at the 0^th^ hour (labelled T = 0 h) and after heating for 72 hours (labelled T = 72 h). For the latter, only the signals corresponding to nitrobenzene (shaded in purple) and formic acid (shaded in tan) can be observed, from which we conclude that the hydrogenation does not proceed unless catalyzed. In the presence of Pt-Melon-M, aniline is formed based on the signals shaded in light blue when using the catalyst with 0.2 wt% platinum loading, though significant quantity of nitrobenzene is still observed as highlighted in purple. For the catalyst with 3 wt% loading, nitrobenzene conversion is largely completed based on the (near-)absence of its signal. In both cases, additional signals shaded in gray are also observed that correspond to neither the two precursors nor to aniline. One possibility is that any aniline formed can condense with formic acid to yield other products, such as formanilide (Figure S28a)ii)).

To verify that aniline can react further and to verify that such reaction(s) proceed without catalyst, we performed a second control whereby aniline and formic acid (but no catalyst) were heated together under identical conditions as the transfer hydrogenation, and the ^1^H NMR spectra (collected in D_2_O) are shown in Figure S28c for the reaction mixture before (black plot, labelled as T = 0 h) and after heating (red plot, T = 72 h). For the former spectrum, only three signals are observed, one for formic acid at 8.3 ppm, two from aniline comprising overlapping triplets at 7.6 ppm and at a doublet at 7.5 ppm. For the latter spectrum, signals in the range 7.7–7.4 ppm became more complex suggesting the presence of aniline together with aniline derivative(s). The additional signals shaded in gray in Figure S28b have chemical shifts similar to those of the additional peaks of the red plot in Figure S28c. One assignment for these peaks is to formanilide as mentioned above, in which case the singlet at 8.6 ppm and the two overlapping doublets at around 7.4–7.2 ppm could be assigned as color-coded in the inset of Figure S28c. Combined, these results demonstrate that the product of nitrobenzene hydrogenation, aniline, can react further with formic acid even without Pt catalyst to yield formanilide and possibly other product(s). Since the focus of this work is on the stability of Pt–gC_3_N_4_ catalysts, detailed analysis of the reaction outcome is beyond the scope here. As such, these yet-to-be verified product(s) from over-reaction of aniline will be subsequently designated simply as “aniline derivatives”. For quantification of the reactant nitrobenzene and the product aniline and its derivative(s), gas chromatography was used, and an example of chromatogram is shown below in Figure S29.

Figure S29 Example of a chromatogram of the reaction mixture after completion of the catalyzed transfer hydrogenation.

In this chromatogram, one can observe the peaks corresponding to aniline (retention time 5.4 min), nitrobenzene (6.8 min) and dodecane as internal standard (7.8 min). Other peaks such as the one with retention time of 8.4 min are considered to be aniline derivatives such as formanilide as mentioned above. The quantitative outcomes of the transfer hydrogenation catalyzed by Pt–gC_3_N_4_ and Pt–TiO_2_ are summarized in Figure S30, which shows the nitrobenzene conversion and product selectivity. Here, the orange columns represent the percentage of nitrobenzene converted, the green columns the amount of aniline, and the purple columns aniline derivatives such as formanilide, whereby the values of the green and purple columns sum to the value of the orange columns within experimental error as indication of mass balance.

Figure S30 Transfer hydrogenation of nitrobenzene using formic acid as hydrogen source as catalyzed by the three Pt–gC_3_N_4_ as compared to Pt–TiO_2_, all having platinum loading of 0.2 wt%. All values shown are averages of triplicates, with error bars representing the standard deviation.

Evidently, all catalysts having 0.2 wt% Pt loading resulted in nitrobenzene conversion in the range of 60–80%, of which the yields of aniline and aniline derivatives were 10–20% and 50–65%. These results demonstrate not only the applicability of the platinized gC_3_N_4_ as catalyst for transfer hydrogenation, but also illustrate this reaction to be a stress test for the stability of these catalytic systems, given that the product aniline (and/or its derivatives) could cause platinum detachment from the support through platinum-amine coordination.

Figure S31 Examples of atomic emission spectra showing the characteristic platinum emission line for the three Pt–gC_3_N_4_ samples after the transfer hydrogenation reaction: a) Pt–Melon-M; b) Pt–Melon-U, and c) Pt–KPHI. Each gray plot corresponds to the supernatant solution after transfer hydrogenation, where the signal signifies platinum leached from the Pt–gC_3_N_4_ into the solution. Each red plot corresponds to the supernatant solution that underwent identical heating (120 °C for 72 h) and has identical composition to that used for transfer hydrogenation but without nitrobenzene (i.e. containing only catalyst, water and formic acid), where the absence of discernible signal signifies the absence of platinum leached from the Pt–gC_3_N_4_. This in turn demonstrates the stability of the gC_3_N_4_ support against decomposition under hot, weakly acidic conditions that would otherwise result in platinum detachment.

REFERENCES

(1) Krause, L.; Herbst-Irmer, R.; Sheldrick, G. M.; Stalke, D. Comparison of silver and molybdenum microfocus X-ray sources for single-crystal structure determination. *J. Appl. Crystallogr.* **2015**, *48*, 3-10

(2) Sheldrick, G. SHELXT - Integrated space-group and crystal-structure determination. *Acta Crystallogr. Sect. A: Found. Crystallogr.* **2015**, *71*, 3-8

(3) Sheldrick, G. Crystal structure refinement with SHELXL. *Acta Crystallogr. Sect. C: Cryst. Struct. Commun.* **2015**, *71*, 3-8

(4) Riggs, W. M. X-ray photoelectron spectrometry of platinum compounds. *Anal. Chem.* **1972**, *44*, 830-832

(5) Contour, J. P.; Mouvier, G.; Hoogewys, M.; Leclere, C. X-ray photoelectron spectroscopy and electron microscopy of Pt–Rh gauzes used for catalytic oxidation of ammonia. *J. Catal.* **1977**, *48*, 217-228

(6) Idriss, H. On the wrong assignment of the XPS O1s signal at 531–532 eV attributed to oxygen vacancies in photo- and electro-catalysts for water splitting and other materials applications. *Surf. Sci.* **2021**, *712*, 121894

(7) Aduru, S.; Contarini, S.; Rabalais, J. W. Electron-, x-ray-, and ion-stimulated decomposition of nitrate salts. *J. Phys. Chem.* **1986**, *90*, 1683-1688

(8) Lau, V. W.-h.; Lu, C.-F.; Putri Wijaya, N.; Lutan, M. Can X-ray Photoelectron Spectroscopy Characterize Photocatalytically Relevant Defects in Graphitic Carbon Nitride? Re-examining Peak Assignment and Quantification Using Model Compounds. *Chem. Mater.* **2024**, *36*, 9762-9774

(9) Kolbeck, C.; Taccardi, N.; Paape, N.; Schulz, P. S.; Wasserscheid, P.; Steinrück, H.-P.; Maier, F. Redox chemistry, solubility, and surface distribution of Pt(II) and Pt(IV) complexes dissolved in ionic liquids. *J. Mol. Liq.* **2014**, *192*, 103-113

(10) Dou, D.; Liu, D.-J.; Williamson, W. B.; Kharas, K. C.; Robota, H. J. Structure and chemical properties of Pt nitrate and application in three-way automotive emission catalysts. *Appl. Catal. B* **2001**, *30*, 11-24

(11) Bancroft, G. M.; Adams, I.; Coatsworth, L. L.; Bennewitz, C. D.; Brown, J. D.; Westwood, W. D. ESCA study of sputtered platinum films. *Anal. Chem.* **1975**, *47*, 586-588

(12) Cattabriga, E.; Ciabatti, I.; Femoni, C.; Funaioli, T.; Iapalucci, M. C.; Zacchini, S. Syntheses, Structures, and Electrochemistry of the Defective ccp [Pt_33_(CO)_38_]^2–^ and the bcc [Pt_40_(CO)_40_]^6–^ Molecular Nanoclusters. *Inorg. Chem.* **2016**, *55*, 6068-6079

(13) Ciabatti, I.; Femoni, C.; Iapalucci, M. C.; Longoni, G.; Zacchini, S. Tetrahedral [HnPt4(CO)4(P∧P)2]n+ (n = 1, 2; P∧P = CH2═C(PPh2)2) Cationic Mono- and Dihydrido Carbonyl Clusters Obtained by Protonation of the Neutral Pt4(CO)4(P∧P)2. *Organometallics* **2013**, *32*, 5180-5189

(14) Chen, Y.; Liu, A.-G.; Chen, Z.-T.; Liang, X.-H.; Yan, J.-T.; Li, B. Efficient IrIII Photosensitizer Incorporated in the Metal–Organic Framework with the Bis-lophine-bipyridine Motif for C(sp3)-C/N–H Cross-Coupling Reactions. *ACS Catal.* **2024**, *14*, 16605-16617

(15) Jones, P. G. Crystal structure determination: a critical view. *Chem. Soc. Rev.* **1984**, *13*, 157-172

(16) Horvath-Bordon, E.; Kroke, E.; Svoboda, I.; Fuess, H.; Riedel, R. Potassium melonate, K_3_[C_6_N_7_(NCN)_3_]·5H_2_O, and its potential use for the synthesis of graphite-like C_3_N_4_ materials. *New J. Chem.* **2005**, *29*, 693

(17) Makowski, Sophia J.; Gunzelmann, D.; Senker, J.; Schnick, W. Protonated Melonate Ca[HC_6_N_7_(NCN)_3_]·7H_2_O - Synthesis, Crystal Structure, and Thermal Properties. *Z. Anorg. Allg. Chem.* **2009**, *635*, 2434-2439

(18) Clauss, C.; Schmidt, H.; Schwarzer, A.; Kroke, E. Copper(II) Melonates Cu_3_[C_6_N_7_(NCN)_3_]_2_·8H_2_O and [Cu(C_2_H_8_N_2_)_2_]_3_[C_6_N_7_(NCN)_3_]_2_·4H_2_O – Using the Terminal Cyano Group of the[C_6_N_7_(NCN)_3_]^3–^ Ion for Complexation. *Z. Anorg. Allg. Chem.* **2011**, *637*, 2246-2251

(19) Clauss, C.; Böhme, U.; Schwarzer, A.; Kroke, E. Silver Melonates and Coordination Modes of the Multidentate [C_6_N_7_(NCN)_3_]^3–^ Anion. *Eur. J. Inorg. Chem.* **2012**, *2012*, 978-986

(20) Makowski, S. J.; Schwarze, A.; Schmidt, P. J.; Schnick, W. Rare-Earth Melonates LnC_6_N_7_(NCN)_3_·xH_2_O (Ln = La, Ce, Pr, Nd, Sm, Eu, Tb; x = 8–12): Synthesis, Crystal Structures, Thermal Behavior, and Photoluminescence Properties of Heptazine Salts with Trivalent Cations. *Eur. J. Inorg. Chem.* **2012**, *2012*, 1832-1839

(21) Makowski, S. J.; Köstler, P.; Schnick, W. Formation of a Hydrogen-Bonded Heptazine Framework by Self-Assembly of Melem into a Hexagonal Channel Structure. *Chem. Eur. J.* **2012**, *18*, 3248-3257

(22) Meng, H.; Meng, P.; Liu, Z.; McMurtrie, J.; Xu, J. Exclusive Coordination between Melem and Silver(I) Ions: From Irregular Aggregates to Nanofibers to Crystal Cubes. *Inorg. Chem.* **2024**, *63*, 6980-6987

(23) Lotsch, B. V.; Döblinger, M.; Sehnert, J.; Seyfarth, L.; Senker, J.; Oeckler, O.; Schnick, W. Unmasking Melon by a Complementary Approach Employing Electron Diffraction, Solid-State NMR Spectroscopy, and Theoretical Calculations — Structural Characterization of a Carbon Nitride Polymer. *Chem. Eur. J.* **2007**, *13*, 4969-4980

(24) Schlomberg, H.; Kröger, J.; Savasci, G.; Terban, M. W.; Bette, S.; Moudrakovski, I.; Duppel, V.; Podjaski, F.; Siegel, R.; Senker, J.; Dinnebier, R. E.; Ochsenfeld, C.; Lotsch, B. V. Structural Insights into Poly(Heptazine Imides): A Light-Storing Carbon Nitride Material for Dark Photocatalysis. *Chem. Mater.* **2019**, *31*, 7478-7486

(25) Choi, M.-J.; Park, H.; Engelhard, M. H.; Li, D.; Sushko, P. V.; Du, Y. Reevaluation of XPS Pt 4f peak fitting: Ti 3s plasmon peak interference and Pt metallic peak asymmetry in Pt@TiO_2_ system. *J. Vac. Sci. Technol. A* **2024**, *42*,

(26) Nguyen, P. A.; Dao, Q. D.; Dang, T. T.; Hoang, T. V. A.; Chung, J. S.; Shin, E. W. Highly dispersed PtO over g-C_3_N_4_ by specific metal-support interactions and optimally distributed Pt species to enhance hydrogen evolution rate of Pt/g-C_3_N_4_ photocatalysts. *Chem. Eng. J.* **2023**, *464*, 142765

(27) Xing, W.; Tu, W.; Ou, M.; Wu, S.; Yin, S.; Wang, H.; Chen, G.; Xu, R. Anchoring Active Pt^2+^/Pt^0^ Hybrid Nanodots on g-C_3_N_4_ Nitrogen Vacancies for Photocatalytic H_2_ Evolution. *ChemSusChem* **2019**, *12*, 2029-2034

(28) Woicik, J. C.; Weiland, C.; Jaye, C.; Fischer, D. A.; Rumaiz, A. K.; Shirley, E. L.; Kas, J. J.; Rehr, J. J. Charge-transfer satellites and chemical bonding in photoemission and x-ray absorption of SrTiO_3_ and rutile TiO_2_: Experiment and first-principles theory with general application to spectroscopic analysis. *Phys. Rev. B* **2020**, *101*, 245119

(29) Diebold, U.; Madey, T. E. TiO_2_ by XPS. *Surf. Sci. Spectra* **1996**, *4*, 227-231

(30) Ocal, C.; Ferrer, S. Low temperature diffusion of Pt and Au atoms through thin TiO_2_ films on a Ti substrate. *Surf. Sci.* **1987**, *191*, 147-156
